# Iontronic Regulation of Nanoconfined Electrical Double Layers on Dielectric Solids

**DOI:** 10.1002/smsc.202500371

**Published:** 2025-09-04

**Authors:** Xiang Li, Yu Wei, Zhong Lin Wang, Di Wei

**Affiliations:** ^1^ Beijing Institute of Nanoenergy and Nanosystems Chinese Academy of Sciences Beijing 101400 P. R. China; ^2^ School of Nanoscience and Engineering University of Chinese Academy of Sciences Beijing 100049 P. R. China

**Keywords:** dynamic regulation, energy harvesting and conversion, information scavenging and modulation, nanoconfined electrical double layer

## Abstract

The electrical double layer (EDL) at solid‐liquid interfaces has been recognized as a pivotal platform for coupling ionic and electronic processes, through which integrated energy harvesting and intelligent information modulation can be enabled. In this review, the evolution from classical EDL models at conductor‐liquid interfaces to extended frameworks for nonconductor‐liquid systems is revisited, and the structure‐function relationships across diverse interfacial environments are elucidated. Building upon this foundation, the implemented energy and information technologies are systematically classified based on the dynamic regulation strategies of different EDL substructures. For energy harvesting and conversion, solid–liquid triboelectric nanogenerators and charge‐supplementary triboiontronics nanogenerators are developed via the regulation of the diffuse layer, while entire‐EDL modulation gives rise to technologies like hydrovoltaic nanogenerators and triboiontronics nanogenerators based on asymmetric EDLs. Moreover, mechanisms for information scavenging and modulation are examined, where diffuse‐layer dynamics are utilized for interfacial charge probe, entire‐EDL reconfiguration is applied to neuromorphic circuit control, and ionic memory emulation, underwater wireless communication, and neuromimetic logic gates are implemented—largely inspired by biological signal transmission. Finally, future application scenarios are outlined, while key challenges are analyzed. Through this comprehensive overview, guidance is provided for leveraging dynamically regulated EDLs to advance next‐generation multifunctional, energy‐information‐coupled systems.

## Graphical abstract

In this review, the evolution from classical EDL models at conductor‐liquid interfaces to extended frameworks for nonconductor‐liquid systems is revisited. Building upon this foundation, the implemented energy and information technologies are systematically classified based on the dynamic regulation strategies of different EDL substructures. Furthermore, future application scenarios are outlined, while key challenges are analyzed.

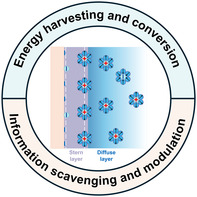

## 1 Introduction

Nanoconfined electrical double layers (EDLs) represent fundamental interfacial architectures that arise at solid–liquid interfaces,^[^ ^1^, ^2^, ^3^, ^4^, ^5^ ^]^ where they mediate a wide spectrum of nanoscale physicochemical processes.^[^ ^6^, ^7^, ^8^, ^9^, ^10^ ^]^ In confined regimes, EDLs critically influence interfacial charge distribution,^[^ ^11^, ^12^, ^13^, ^14^, ^15^, ^16^ ^]^ ion transport,^[^ ^17^, ^18^, ^19^, ^20^ ^]^ electrochemical reactivity,^[^ ^21^, ^22^, ^23^, ^24^, ^25^ ^]^ and ultimately, macroscopic properties such as energy storage capacity,^[^ ^26^, ^27^, ^28^, ^29^ ^]^ charge‐transfer kinetics,^[^ ^30^, ^31^, ^32^, ^33^ ^]^ interfacial wettability,^[^ ^34^, ^35^ ^]^ and catalytic activity.^[^ ^36^, ^37^, ^38^, ^39^ ^]^ Over nearly two centuries, the scientific community has endeavored to elucidate the structure and behavior of EDLs, leading to the establishment of three foundational models at conductor‐liquid interfaces. The Helmholtz model^[^ ^40^ ^]^ conceptualizes the EDL as a compact capacitor‐like layer, the Gouy–Chapman model^[^ ^41^, ^42^ ^]^ introduces a thermally distributed diffuse layer, and the Gouy–Chapman–Stern (GCS) model^[^ ^43^ ^]^ integrates both compact Stern layer and diffuse layer to provide a more realistic representation of ionic distributions near conductive surfaces.^[^ ^44^, ^45^ ^]^ Among these, the GCS model remains the most widely adopted framework in electrochemistry^[^ ^46^, ^47^, ^48^, ^49^, ^50^ ^]^ and interfacial science.^[^ ^51^, ^52^, ^53^, ^54^ ^]^ Despite their historical significance, classical EDL models inherently presume the presence of free charge carriers within the solid phase, rendering them applicable primarily to conductor‐liquid interfaces.^[^ ^55^, ^56^, ^57^, ^58^ ^]^ However, many emerging applications involve dielectric (non‐conductive) solids, such as ranging from nanogenerators^[^ ^59^, ^60^, ^61^, ^62^, ^63^ ^]^ and biosensors^[^ ^64^, ^65^, ^66^, ^67^ ^]^ to soft robotics^[^ ^68^, ^69^, ^70^, ^71^ ^]^ and neuromorphic systems,^[^ ^72^, ^73^, ^74^, ^75^ ^]^ where the absence of mobile carriers leads to fundamentally different interfacial charge behaviors. In such systems, traditional models fail to capture the complex interplay between induced surface polarization, ionic rearrangement, and nonequilibrium effects. As a result, there is a pressing need to develop new conceptual frameworks capable of describing EDL formation and dynamics at dielectric‐liquid interfaces, particularly under nanoconfinement.

To address this theoretical gap, recent advances in high‐resolution interfacial characterization have enabled a more direct investigation of EDL formation on dielectric materials. Notably, Wang's group employed Kelvin Probe Force Microscopy (KPFM) to reveal the coexistence of surface‐bound electrons and mobile ions at the dielectric‐liquid interface,^[^ ^76^, ^77^ ^]^ challenging the ion‐only paradigm of classical models. These findings led to the formulation of a two‐step EDL model at nonconductor‐liquid interfaces, wherein the double layer forms through a sequential process:^[^ ^76^, ^77^, ^78^, ^79^ ^]^ i) an initial polarization stage involving electron cloud overlap and interfacial ionization, generating an electronic‐ionic polarization layer, followed by ii) the subsequent redistribution of ions to compensate the induced potential. This model provides a more accurate depiction of charge organization at dielectric surfaces, yet it remains inherently passive, restricted by thermodynamic equilibrium and lacking dynamic tunability. In response, the emerging triboiontronics EDL model at nonconductor‐liquid interfaces introduces a new paradigm of active EDL regulation through contact electrification and triboelectric‐induced polarization.^[^ ^80^, ^81^, ^82^ ^]^ This approach enables real‐time control over interfacial ionic‐electronic coupling using mechanical inputs, transforming traditionally passive interfaces into programmable platforms for energy harvesting and information processing.^[^ ^83^, ^84^, ^85^, ^86^, ^87^ ^]^ The triboiontronics EDL model thus extends beyond equilibrium thermodynamics, leveraging external mechanical stimuli to dynamically modulate EDL structure, charge density, and transport properties. More importantly, the synergy of ionic and electronic charge carriers within this framework gives rise to iontronic systems as a new class of devices capable of efficiently managing both energy and information flows. Compared to conventional electronics, which rely solely on electrons, iontronics allows for the co‐transport and coupling of high‐valence ions and electrons at the solid‐liquid boundary, offering a versatile alternative for applications in energy harvesting, adaptive sensing, and neuromorphic control. Despite these promising advances, the field currently lacks a cohesive understanding of the mechanisms, device architectures, and functional potential enabled by dynamically regulated EDLs on dielectric materials.

**Figure 1:**
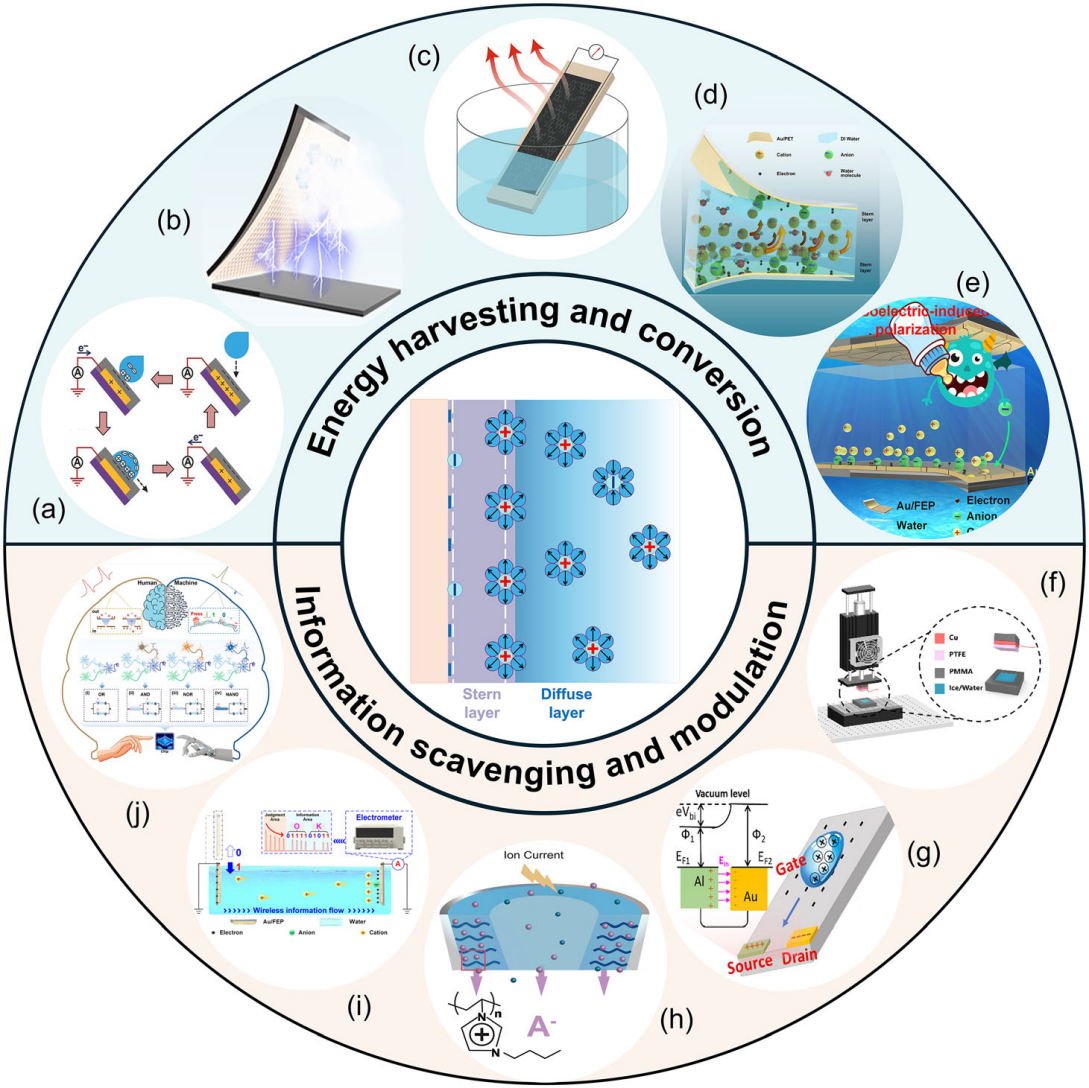
The dynamic regulation of distinct EDL substructures was categorized, and their implications for energy harvesting and conversion, and information acquisition and modulation were systematically explored. In the energy domain, the regulation of the diffuse layer led to devices. a) SL‐TENGs. Reproduced with permission.^[^ ^88^ ^]^ Copyright 2014, WILEY‐VCH Verlag GmbH & Co. KGaA, Weinheim. b) CS‐TINGs. Reproduced with permission.^[^ ^80^ ^]^ Copyright 2023, Elsevier Inc. Whole‐EDL modulation gave rise to technologies. c) Hydrovoltaic nanogenerators. Reproduced with permission.^[^ ^93^ ^]^ Copyright 2018, The Author(s). d) TINGs based on asymmetric EDLs. Reproduced with permission.^[^ ^98^ ^]^ Copyright 2024, The Author(s). e) TINGs based on asymmetric EDLs enhanced by triboelectric‐induced polarization. Reproduced with permission.^[^ ^99^ ^]^ Copyright 2025, Elsevier Inc. In the information domain, regulation of the diffuse layer could enable EDL‐based interface probes. Reproduced with permission.^[^ ^100^ ^]^ Copyright 2024, Elsevier Ltd. More comprehensive regulation of the entire EDL has allowed for information modulation technologies. g) Bioinspired neuromorphic circuit control. Reproduced with permission.^[^ ^103^ ^]^ Copyright 2024, The Author(s). h) Ionic memory for synaptic emulation. Reproduced with permission.^[^ ^106^ ^]^ Copyright 2023, The American Association for the Advancement of Science. i) Underwater wireless information transmission. Reproduced with permission.^[^ ^99^ ^]^ Copyright 2025, Elsevier Inc. j) The neuromimetic logic gate driven by ionic‐electronic coupling was designed. Reproduced with permission.^[^ ^109^ ^]^ Copyright 2025, Wiley‐VCH GmbH.

In this review, a comprehensive and integrative framework was established to understand the structural evolution and iontronic regulation of EDLs at dielectric‐liquid interfaces. The theoretical progression of EDL models was first revisited, from the classical GCS model describing conductor–liquid interfaces, to the passive two‐step framework applicable to nonconductor‐liquid systems, and ultimately to the actively reconfigurable triboiontronics paradigm tailored for nonconductive interfaces. The underlying mechanisms governing charge formation, interfacial polarization, and coupled ionic‐electronic transport were elucidated. Particular attention was given to how each model has successively expanded the understanding of charge dynamics at solid–liquid boundaries, especially in systems without intrinsic electronic conductivity. Subsequently, dynamic regulation strategies targeting distinct EDL substructures were categorized, and their practical implications were systematically examined in two major domains: energy harvesting and conversion, and information acquisition and modulation (**Figure** **1**). In the energy domain, EDL‐based systems were classified according to the region being regulated: Modulation of the diffuse layer was employed in devices such as solid–liquid triboelectric nanogenerators (SL‐TENGs)^[^ ^59^, ^88^, ^89^, ^90^, ^91^, ^92^ ^]^ and charge‐supplementary triboiontronic nanogenerators (CS‐TINGs),^[^ ^80^ ^]^ whereas modulation of the entire EDL was realized in hydrovoltaic nanogenerators^[^ ^93^, ^94^, ^95^, ^96^, ^97^ ^]^ and TINGs based on asymmetric EDLs.^[^ ^98^, ^99^ ^]^ Distinct output behaviors were exhibited across these categories to meet diverse application requirements. In the context of information technologies, diffuse‐layer regulation has been utilized to enable sensitive probing of interfacial charge variations, allowing for the development of EDL‐based sensing interfaces.^[^ ^14^, ^100^, ^101^ ^]^ More comprehensive modulation of the entire EDL has been applied to construct bioinspired signal‐processing systems, including neuromorphic circuit control,^[^ ^80^, ^102^, ^103^ ^]^ ionic memory for synaptic emulation,^[^ ^104^, ^105^, ^106^ ^]^ underwater wireless information transmission,^[^ ^99^, ^107^, ^108^ ^]^ and neuromimetic logic gates governed by ionic‐electronic coupling.^[^ ^109^ ^]^ In these systems, dynamic EDL regulation is exploited to bridge ionic and electronic signal domains for multifunctional transduction driven by mechanical stimuli. Finally, application scenarios are projected in fields such as self‐regulating energy harvesting, biomechanical energy conversion, and bio‐interfaced neuromorphic platforms. Meanwhile, key challenges are identified and analyzed, including limitations in spatial precision, interfacial instability, and a lack of unified frameworks for co‐optimizing energy and information flow. Through the unification of these diverse research trajectories, a coherent roadmap is proposed for the realization of next‐generation adaptive, multifunctional iontronic platforms driven by dynamically regulated EDLs at dielectric‐liquid interfaces.

## 2 Typical EDL Models at Different Solid–Liquid Interfaces

The structural understanding of EDLs at conductor‐liquid interfaces has historically been framed through a series of progressively refined theoretical models, each capturing different aspects of ion distribution and interfacial electrostatics. The earliest conceptualization, known as the Helmholtz model (1879),^[^ ^40^ ^]^ depicted the EDL as a rigid, capacitor‐like structure consisting of a fixed layer of counterions adsorbed at the liquid side, directly opposing an equally dense layer of surface charges on the solid electrode. Although this model provided the first approximation of EDL‐induced interfacial potential drop, it assumed a fixed spatial arrangement of ions and failed to account for thermal motion or ionic diffusion, thus limiting its ability to describe dynamic or diffuse interfacial behavior. To address these limitations, the Gouy–Chapman model was developed in the early 20th century,^[^ ^41^, ^42^ ^]^ introducing the concept of a diffuse layer of counterions governed by Boltzmann statistics and electrostatic interactions. This model conceptualized the liquid‐side ionic distribution as a continuous gradient extending from the electrode surface into the bulk solution, with ion concentration exponentially decaying with distance from the interface. While more realistic in capturing thermal and entropic effects, the Gouy–Chapman formulation significantly overestimated capacitance at high surface potentials and failed to incorporate finite ion size or specific adsorption effects near the interface.

**Figure 2:**
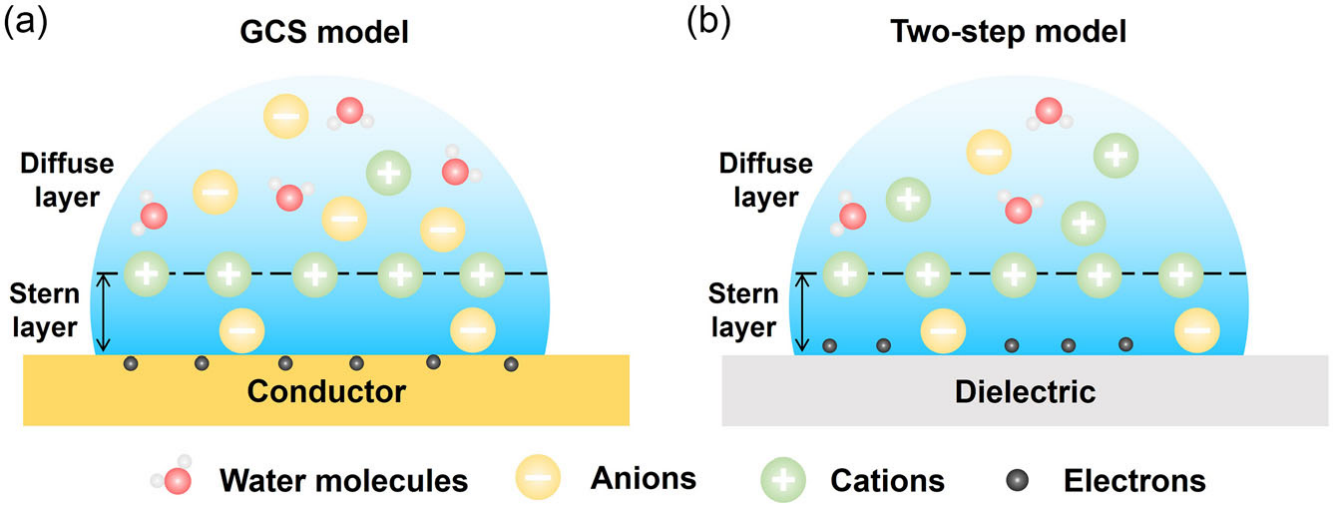
Typical EDL models at different solid‐liquid interfaces. a) The GCS model at conductor‐liquid interfaces. b) The GCS model at non‐conductor‐liquid interfaces.

To reconcile these discrepancies, the GCS model was subsequently proposed as a hybrid framework,^[^ ^43^ ^]^ integrating the strengths of both previous models (**Figure** **2**a). In the GCS model, the EDL is divided into two discrete regions: a compact Stern layer, adjacent to the electrode surface, where specifically adsorbed ions and oriented solvent molecules form a relatively immobile, high‐field region; and an adjacent diffuse layer, where counterions are distributed according to the Gouy–Chapman profile. This bipartite architecture effectively captures both the electrostatic screening of mobile ions and the interfacial structuring due to specific ion‐surface interactions. The GCS model has since become the foundational paradigm for describing EDLs at conductor‐liquid interfaces, underpinning the theoretical basis for a wide array of interfacial processes, including electrocatalysis, electrochemical energy storage, and surface charge regulation. Critically, the GCS model provides a framework through which differential capacitance, surface potential, and ionic distribution can be quantitatively predicted and experimentally validated. Its modular structure also enables integration with more advanced theories incorporating ion correlation, dielectric saturation, or specific adsorption kinetics. As such, the GCS model continues to serve as a cornerstone for understanding interfacial charge phenomena at conductor‐liquid boundaries. However, the assumption of electronic conductivity in the solid phase limits its applicability to nonconductive (dielectric) interfaces, necessitating the development of alternative models to capture EDL behavior in such systems.

While the GCS model has provided a robust framework for understanding EDL behavior at conductive interfaces, it becomes inadequate when extended to nonconductive solids, which inherently lack mobile charge carriers within the bulk phase. At such interfaces, conventional assumptions such as free electron redistribution or direct electrochemical interaction no longer hold. In response to this conceptual gap, a two‐step EDL model has been proposed by Wang's group to describe the charge formation and distribution processes unique to nonconductor‐liquid interfaces,^[^ ^1^, ^76^ ^]^ particularly under nanoconfined or contact‐induced conditions (Figure 2b). This model was empirically motivated by a series of surface potential and charge mapping studies. In particular, Wang's group employed KPFM to directly probe the potential landscape at the interface between dielectric substrates (e.g., SiO_2_) and various aqueous phases.^[^ ^76^, ^78^ ^]^ As reported, a series of charging‐heating cycle experiments was performed using SiO_2_ substrates in contact with aqueous solutions. After CE with deionized water, the total surface charge density reached −810 μC m^−2^. Upon heating the sample to 513 K, approximately −630 μC m^−2^ of this charge decayed rapidly in accordance with thermionic emission behavior, corresponding to mobile electronic charge release. The remaining −180 μC m^−2^ persisted through thermal cycling and accumulated over successive contacts, indicating the presence of immobile, covalently bound ionic species, primarily *O* ^−^ generated by hydroxyl surface ionization. This partitioning suggests that ≈77% of the initial interfacial charge arose from electronic polarization, while ≈23% was due to ionic compensation in this specific system. Furthermore, those studies systematically explored the influence of surface wettability on the electron‐to‐ion transfer ratio. Surfaces with lower water contact angles (i.e., more hydrophilic) showed higher ion transfer contributions, whereas more hydrophobic surfaces favored electronic charge accumulation. For instance, AlN (water contact angle ≈97°) exhibited over 88% electronic charge contribution, while Si3N4 (water contact angle ≈40°) was dominated by ionic species. These trends underscore the importance of the interfacial environment in modulating charge carrier types and densities. Their findings revealed a complex interfacial structure characterized not only by the presence of mobile ions from the liquid phase, but also by localized electronic polarization and induced surface‐bound charges. These observations indicated that dielectric interfaces may participate in more intricate charge compensation mechanisms involving electron cloud overlap, surface dipole rearrangement, and non‐Faradaic interfacial reactions.

Based on these findings, the two‐step EDL model was formulated as shown in Figure 2b.^[^ ^1^, ^76^ ^]^ In this framework, charge formation is considered to proceed via two sequential processes. In the first stage, upon contact between the dielectric solid and the liquid, electronic polarization is first induced within the solid due to electron cloud overlap and potential hydrogen bonding interactions at the interface. Simultaneously, partial ionization of surface groups (e.g., ‐OH, ‐F, or surface‐adsorbed water molecules) may occur, producing localized fixed charges. Therefore, the inner Helmholtz plane (IHP) is formed, which is composed of electrons and ions. In the second stage, following primary polarization, mobile ions from the liquid phase redistribute to electrostatically compensate the surface‐bound charges to form the outer Helmholtz plane (OHP), thereby forming the complete Stern layer. Extending from the Stern layer into the bulk, the diffuse layer is formed in which the distribution of ions is controlled by the balance of electrostatic and entropic forces. However, unlike in conductor‐based models, the overall EDL structure is anchored by an electrostatically asymmetric and partially immobile polarization layer rather than by mobile surface electrons. This model emphasizes the coupled nature of electron and ion behaviors at dielectric‐liquid interfaces and explicitly distinguishes between intrinsic (bound) and extrinsic (mobile) contributions to surface charge. It accounts for the fact that ionic compensation is driven by polarization‐induced fields, rather than by externally imposed electrode potentials or intrinsic conductivity, as is the case in metallic systems. Importantly, for the first step (electron transfer), charge separation is governed by quantum‐mechanical tunneling during transient molecular collisions. Pump‐probe spectroscopy was used to directly observe interfacial electron transfer occurring within 100 ps,^[^ ^110^ ^]^ and complementary first‐principles calculations further confirmed this time scale.^[^ ^111^ ^]^ This ultrafast process is several orders of magnitude faster than the dielectric relaxation time of bulk water (≈1 μs), indicating that electron transfer occurs essentially instantaneously on the scale of any realistic mechanical stimulus. For the second step (ionic polarization and redistribution), the dynamics are governed by field‐driven diffusion within the nanoconfined interfacial region. Reported values from electrochemical impedance spectroscopy measurements in conventional nanochannels suggest characteristic timescales ranging from ≈1 μs to 1 ms,^[^ ^112^ ^]^ depending on ionic mobility and confinement geometry. These timescales are comparable to the dielectric relaxation times of electrolytes, validating the physical consistency of the second step with classical nanofluidic and EDL models. Taken together, these estimates indicate that the full two‐step process operates well within the dynamic range of most practical mechanical actuation. Crucially, the picosecond electron transfer step remains instantaneous in the vast majority of states, ensuring that the fundamental charge separation mechanism remains valid for most of the actuation frequency.

**Figure 3:**
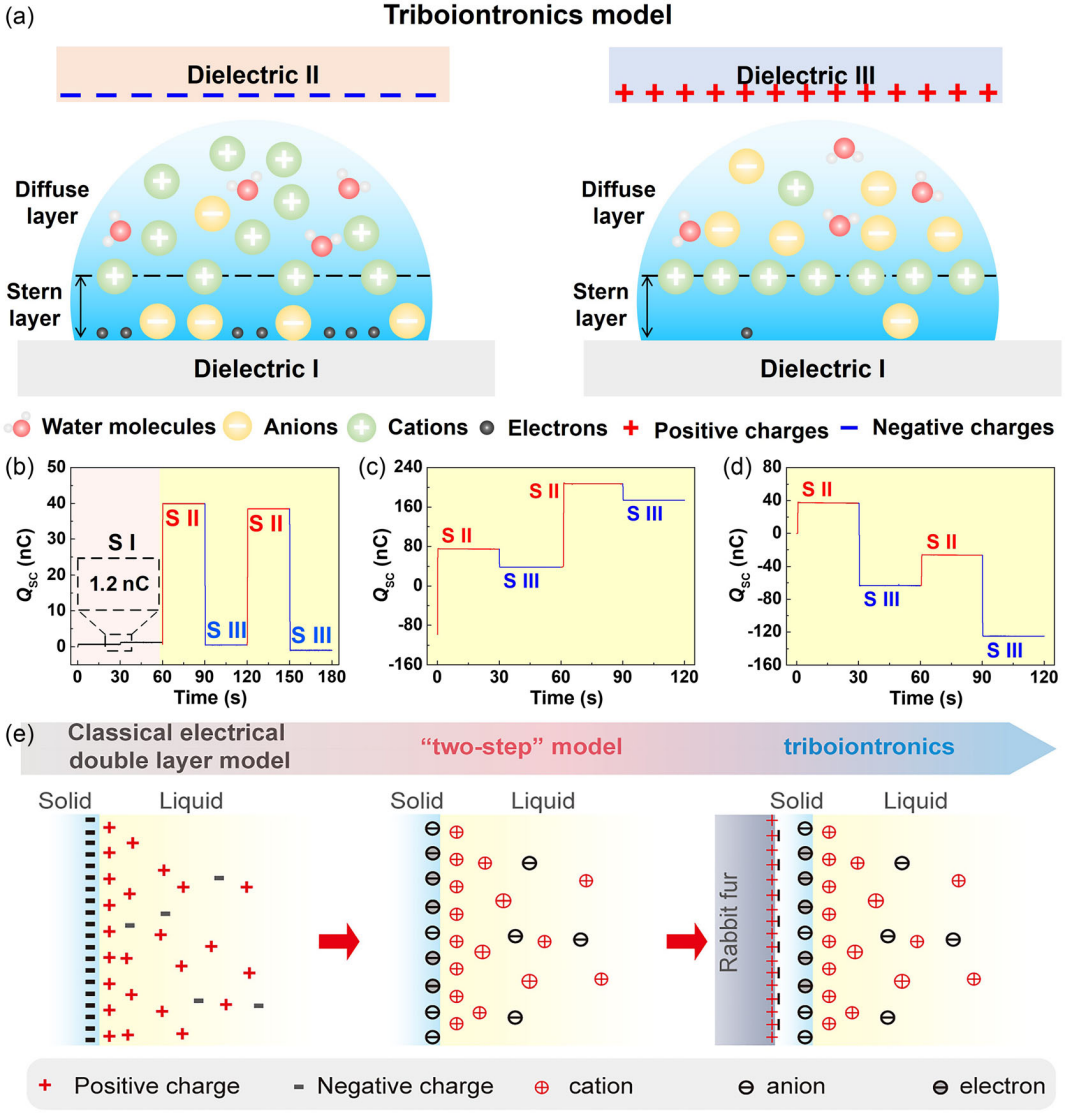
The triboiontronics EDL model at dielectric‐liquid interfaces was proposed by Wei et al. in 2023. a) In this model, triboelectric‐induced polarization is employed as the primary mechanism for tuning the local interfacial potential, thereby regulating the structure and composition of the EDL in real time. b–d) Experimental verification of the EDL structure dynamically regulated by triboelectric‐induced polarization, Reproduced with permission.^[^ ^80^ ^]^ Copyright 2023, Elsevier Inc. e) Wang and colleagues elaborated on the triboiontronics framework originally introduced by Wei et al. in 2023, Reproduced with permission.^[^ ^114^ ^]^ Copyright 2023, Elsevier Inc.

Despite the conceptual advancement offered by the two‐step EDL model in describing interfacial charge formation at dielectric‐liquid interfaces, its applicability remains largely confined to thermodynamically stable or quasi‐static conditions. However, in realistic operational environments, dielectric‐liquid interfaces are frequently subjected to dynamic mechanical or electrostatic perturbations, which introduce nonequilibrium charge redistribution and time‐dependent electrostatic asymmetry. To account for such behavior, the triboiontronics EDL model as a new theoretical framework at dielectric‐liquid interfaces was proposed by Wei et al. in 2023^[^ ^80^, ^81^, ^82^ ^]^ (**Figure** **3**a), which incorporated active modulation of ionic‐electronic coupling interactions under external mechanical stimuli, particularly those derived from contact electrification processes. In this model, triboelectric‐induced polarization is employed as the primary mechanism for tuning the local interfacial potential, thereby regulating the structure and composition of the EDL in real time. Unlike externally biased electrochemical systems, the triboiontronics approach enables self‐driven, material‐intrinsic control over interfacial charge dynamics without the need for applied voltage. On the one hand, the negative surface potential of dielectric II, a forward triboelectric‐induced polarization process is triggered. It not only facilitates the extraction of cations from nearby water molecules to form negatively charged sites near the dielectric I surface, but also promotes the adsorption of lower hydration energy anions onto the dielectric I surface, thereby forming a stronger negatively charged Stern layer and a diffuse layer rich in cations. On the other hand, when the dielectric III surface holds a positive triboelectric charge, an opposite triboelectric‐induced polarization process is triggered. The local field repels cations near the dielectric I surface and attracts anions near the dielectric III surface. However, due to their high hydration enthalpy, cations often fail to penetrate the IHP and instead accumulate near the OHP. The IHP in this case primarily consists of oriented water dipoles, resulting in a net positive potential within the Stern layer and a corresponding enrichment of anions in the diffuse layer. This reversal of interfacial charge polarity under different triboelectric‐induced polarizations gives rise to a bistable EDL architecture, enabling reversible and programmable modulation of surface charge profiles. Therefore, by circumventing the need for conductive substrates or external power sources, the triboiontronics EDL model offers an energy‐efficient and highly tunable strategy for modulating interfacial charge phenomena. Moreover, surface wettability is a key determinant of charge transfer behavior and output performance in solid–liquid systems.^[^ ^113^ ^]^ In the context of dynamic EDL reconfiguration, wettability plays a dual and nonmonotonic role, with both hydrophilic and superhydrophobic extremes introducing distinct limitations. On the one hand, excessively hydrophilic surfaces promote strong water adhesion and dense interfacial contact, enabling the formation of highly stable and compact electrical double layers. However, such stability comes at the cost of reduced tunability: The strongly adsorbed ions in the Stern layer become more difficult to perturb or reconfigure via external electrostatic stimuli. Stronger triboelectric‐induced polarization fields are required to modulate these tightly bound ionic layers, reducing the responsiveness and efficiency of triboiontronic control under mild actuation. On the other hand, superhydrophobic surfaces, while potentially enhancing electronic charge transfer, significantly hinder the formation of robust EDL. The presence of entrapped air pockets and limited solid–liquid contact area (characteristic of the Cassie–Baxter regime) disrupts the EDL and suppresses effective ion adsorption. These factors collectively diminish the ability of the EDL to form, evolve, and respond under dynamic polarization, thereby weakening ionic modulation and signal output. Taken together, neither extreme hydrophilicity nor superhydrophobicity is ideal for achieving efficient and reversible EDL control. Instead, intermediate wettability could offer a more favorable balance: sufficient ionic contact to form a responsive EDL, while maintaining enough interfacial mobility to allow tunable polarization‐driven reconfiguration. Furthermore, the efficiency of dynamically regulating EDL is significantly influenced by the physicochemical properties of the dielectric materials involved. In particular, surface electronegativity plays a critical role in determining the structure, stability, and tunability of the solid–liquid EDL. Dielectric materials with high electronegativity, such as polytetrafluoroethylene (PTFE), fluorinated ethylene propylene (FEP), and SiO_2_, exert a stronger binding force on interfacial electrons, leading to the formation of more compact and tightly bound EDLs. These stabilized layers possess higher charge densities but exhibit slower dynamic responses, thus requiring stronger external polarization fields (i.e., greater triboelectric potential) to achieve effective modulation. In contrast, materials with moderate electronegativity form more loosely bound EDLs that are more readily reconfigured under weaker triboelectric stimuli, enabling faster and more energy‐efficient control of interfacial ionic polarity.

To illustrate this dynamic regulation mechanism, a three‐stage experiment was designed,^[^ ^80^ ^]^ which used a polyethylene terephthalate (PET)‐based humidity generator (spray volume ≈1 mL) to emulate triboiontronics regulation of interfacial ion distributions, as shown in Figure 3b. In Stage I, charged water mist was generated solely through contact electrification between the PET shell and water. The measured transferred charge (*Q*_SC_) of the charged water mist was ≈+1.2  nC, indicating weak interfacial polarization and limited ionic asymmetry. This served as the baseline (quasi‐neutral EDL configuration). In Stage II, a positively charged rabbit fur, prepolarized by contact electrification with a PTFE film, was brought near the PET surface, inducing forward triboelectric‐induced polarization. This external electrostatic field enhanced anion adsorption on the PET surface while promoting cation enrichment in the water mist, resulting in a sharp increase in *Q*_SC_ to + 40.0  nC. The ionic polarity and concentration profile were thus actively reconfigured, demonstrating one stable EDL state characterized by cation‐dominated ion flux. In Stage III, the fur was removed, and electrons were transferred from the fur to the PET surface, reversing the surface potential and thereby inducing reverse triboelectric‐induced polarization. This reversed the interfacial electric field, promoting cation adsorption and enriching anions in the mist. Consequently, the measured *Q*_SC_ flipped to approximately −40.0  nC, clearly demonstrating polarity inversion of the ion transport, and establishing a second, oppositely polarized EDL configuration. Furthermore, in Figure 3c,d, the magnitude and direction of the ion flux can be programmably tuned by adjusting the surface charge density by varying the contact electrification strength. For example, stronger forward triboelectric‐induced polarization resulted in a *Q*_SC_ of +180  nC, indicating an order‐of‐magnitude increase in cation flux. Conversely, enhanced reverse triboelectric‐induced polarization of the PET surface drove a larger anion flux, generating *Q*_SC_ values up to −100  nC. These results confirm not only the bistability of the EDL states but also their continuous tunability via triboelectric actuation. These experimental results provide direct, quantitative evidence that triboelectric‐induced polarization can reversibly and programmably modulate the charge structure of the Stern layer within the EDL, thereby dynamically adjusting the charge polarity and amount in the diffuse layer. Moreover, bistable EDL states are achieved by reversibly switching the polarity of triboelectric‐induced interfacial polarization, which leads to stable and distinct ion distributions at the solid–liquid interface. The system remains in a given polarization state for prolonged periods (typically several tens of seconds) after the removal of the triboelectric trigger, even under ambient thermal noise (Figure 3b–d). This observation implies that the system's bistable states are not spontaneously disrupted by thermal fluctuations at room temperature, indicating the presence of a robust potential barrier between states. Moreover, these states exhibit nonvolatile retention: The surface potential and ionic polarity remain unchanged unless actively reprogrammed by an oppositely charged triboelectric stimulus. This behavior further supports the conclusion that transitions between bistable configurations require overcoming a significant energetic threshold, exceeding the thermal noise floor. Furthermore, the energy barrier could be quantified by analyzing the effective potential difference between states and the corresponding charge, and comparing this with the thermal fluctuations to rigorously assess thermal stability and retention time. More importantly, while the dynamic nature of the EDL at solid–liquid interfaces poses significant experimental challenges, several advanced techniques have emerged as promising tools for probing such processes. In future work, KPFM and surface‐enhanced Raman scattering‐based methods could be combined to establish a spatial‐temporal map of interfacial potential and ionic rearrangement under dynamic triboiontronic control. These techniques will allow us to directly correlate mechanical input with EDL state evolution, further reinforcing the mechanistic foundations of the proposed system.

The triboiontronics EDL model thus represents a substantial conceptual shift from both classical and two‐step formulations by emphasizing nonequilibrium and stimulus‐responsive interfacial dynamics. Mechanistically, it underscores the coupled interaction between triboelectric‐induced polarization (arising on the dielectric surface) and ionic redistribution (existing in the liquid near the contact interface), forming an integrated ionic‐electronic feedback loop. This coupling not only enables the spontaneous generation of interfacial potential but also facilitates dynamic tuning of charge carrier polarity, distribution, and transport pathways, which are features essential for advanced energy and information technologies. In 2023, Wang and colleagues elaborated on the triboiontronics framework originally introduced by Wei et al., emphasizing how real‐time regulation of the EDL can serve as a pivotal mechanism for next‐generation interface design, bio‐interfacing sensors, and interactive human‐machine platforms^[^ ^114^ ^]^ (Figure 3e). In conclusion, the triboiontronics EDL model establishes a mechanically addressable charge control framework at dielectric‐liquid interfaces, capable of modulating the structure, polarity, and dynamics of EDLs with high temporal and spatial precision. By integrating concepts from contact electrification, polarization theory, and interfacial ionics, it lays the groundwork for a new generation of intelligent, self‐powered, and multifunctional iontronic platforms. Accordingly, triboiontronics emerges as a unifying platform for integrating energy conversion and information modulation, driving the advancement of multifunctional and adaptive electronic systems.

## 3 Energy Harvesting and Conversion Based on Dynamic Regulation of the EDL

Following the establishment of triboiontronics mechanisms as a robust framework for interfacial charge manipulation, increasing research attention has been directed toward exploiting the dynamic tunability of the EDL to enhance energy transduction at dielectric‐liquid interfaces. Rather than treating the EDL as a static electrochemical boundary, emerging strategies have emphasized its real‐time structural reconfiguration under mechanical, electrostatic, or chemical perturbations, enabling active modulation of ionic‐electronic coupling and more efficient interfacial energy conversion. From a mechanistic standpoint, EDL‐regulated energy harvesting and conversion systems can be classified according to the specific EDL substructures targeted for modulation. In configurations primarily involving the diffuse layer, such as SL‐TENGs, transient electrostatic fields arising from the Stern layer on the dielectric surface drive reversible ionic displacement in the outer EDL region, producing AC displacement signals. In contrast, CS‐TINGs utilize the ion concentration gradient generated by modulating the local charge density within the diffuse layer to induce directional ionic‐electronic coupling, resulting in a DC output with enhanced power density. More comprehensive approaches aim to dynamically regulate the entire EDL. For example, hydrovoltaic nanogenerators exploit asymmetric ion movement along a moving EDL boundary, typically producing low‐amplitude constant signals. Meanwhile, TINGs based on asymmetric EDLs are capable of inducing DC outputs with higher current amplitudes, owing to the persistent interfacial asymmetry and sustained ion concentration gradients. Importantly, these systems exhibit a wide range of tunable output characteristics, depending on the spatial configuration, temporal responsiveness, and symmetry of EDL modulation. This versatility renders dynamically reconfigurable EDL‐based platforms highly adaptable to a variety of application‐specific energy harvesting and conversion scenarios.^[^ ^115^, ^116^, ^117^, ^118^, ^119^, ^120^, ^121^, ^122^ ^]^

### 3.1 Energy Technologies Based on the Dynamic Regulation of the Diffuse Layer

#### 3.1.1 Solid–Liquid Triboelectric Nanogenerators (SL‐TENGs)

The diffuse layer, representing the outermost region of the EDL, plays a pivotal role in mediating ion transport and interfacial charge dynamics. Unlike the compact Stern layers, where charge localization dominates, the diffuse layer supports mobile ion redistribution under relatively slight external perturbations. Dynamic regulation of this region can therefore enable real‐time manipulation of ion displacement and electric field formation, making it highly suitable for energy transduction applications where mechanical or flow‐induced stimuli are present. In energy harvesting systems based on targeting the diffuse layer, SL‐TENGs represent a key class of devices that harness the interaction between solid surfaces and flowing or oscillating liquids to produce AC output.^[^ ^123^, ^124^, ^125^, ^126^, ^127^, ^128^, ^129^, ^130^ ^]^ By leveraging triboelectric‐induced polarization at the solid‐liquid interface and modulating ion motion within the diffuse layer, SL‐TENGs achieve efficient mechanical‐to‐electrical energy conversion without requiring external bias or conductive electrodes.

**Figure 4:**
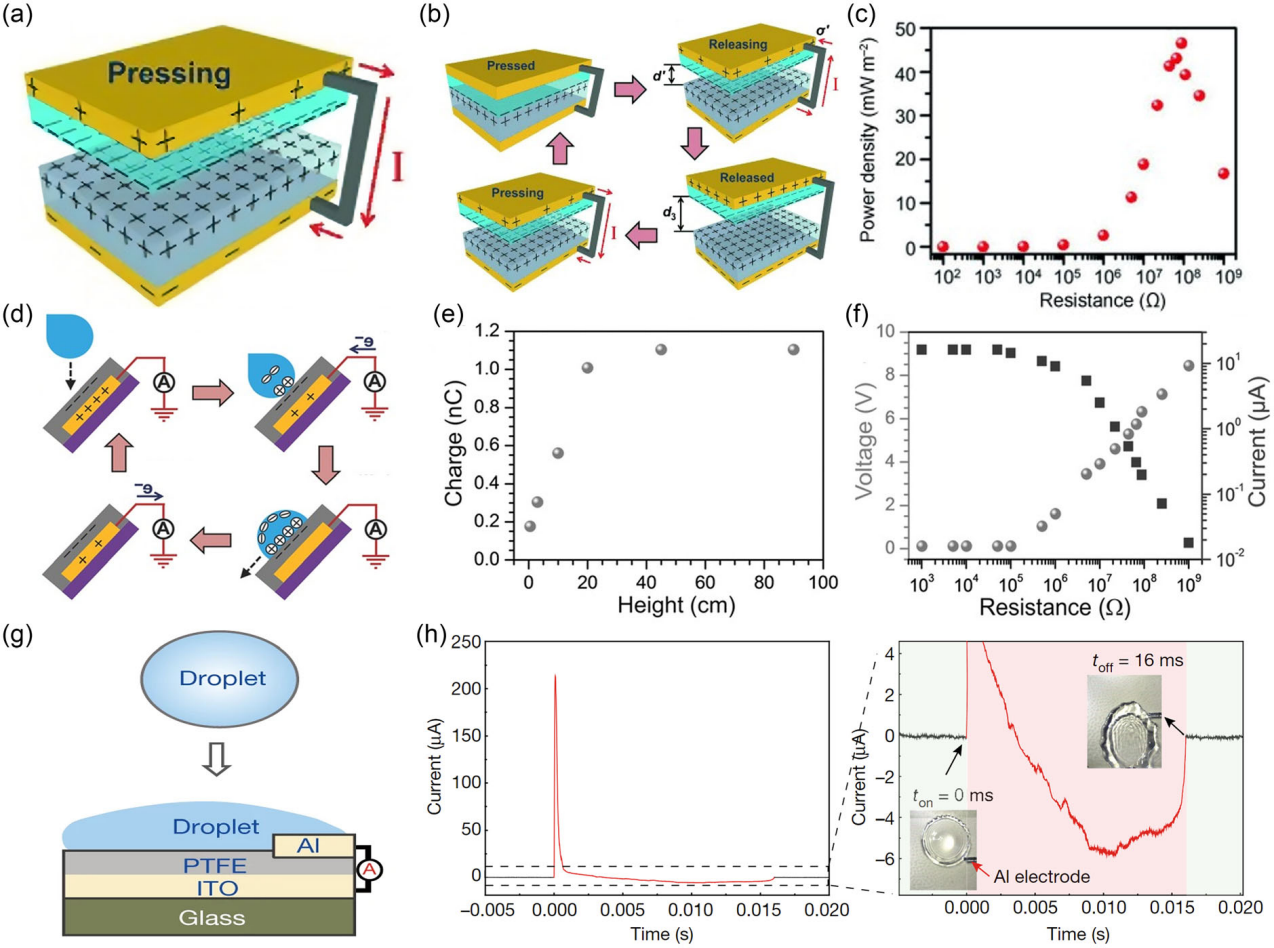
Development and evolution of typical SL‐TENGs and their output characteristics. a) The first SL‐TENG was reported by Wang's group in 2013. Reproduced with permission.^[^ ^89^ ^]^ Copyright 2013, WILEY‐VCH Verlag GmbH & Co. KGaA. b) Demonstration of the operating principle. c) Its *P*_R_ density was nearly 0.13 W/m^2^. d) To expand the application of the SL‐TENG in the field of droplet energy harvesting, a refined structure was introduced by Lin et al. in 2014. Reproduced with permission.^[^ ^88^ ^]^ Copyright 2014, WILEY‐VCH Verlag GmbH & Co. KGaA. e) As the droplet fell from a higher height, the impact force on the interface increased, resulting in more charge transfer. f) The *V*_OC_ and *I*_SC_ were moderate at 9.3 V and 17 μA, respectively. g) A pivotal advancement emerged with the introduction of a DEG proposed by Xu et al. in 2020.^[^ ^131^ ^]^ Copyright 2020, The Author(s), under exclusive license to Springer Nature Limited. h) It exhibited a substantial performance enhancement attributed to a newly identified “bulk effect.”

The first SL‐TENG,^[^ ^89^ ^]^ reported by Wang's group in 2013, demonstrated the feasibility of harvesting energy from periodic water‐solid contact (**Figure** **4**a). The device employed a microstructured polydimethylsiloxane (PDMS) layer as the dielectric layer with regularly spaced pyramidal patterns bonded to a copper (Cu) electrode. Immersed in water, the PDMS film underwent cyclic contact separation driven by mechanical vibration, mimicking wave conditions. The energy generation relied on the dynamic migration of the diffuse layer in the EDL, driven by the interaction between the PDMS film and water (Figure 4b). Upon the first contact, the negatively charged Stern layer was retained on the dielectric, while the adjacent diffuse layer in the liquid acquired net positive charges, forming an EDL. As separation occurred, the EDL structure was disrupted, inducing an ion concentration gradient in the diffuse layer to drive ion migration, thereby generating a potential difference in the external circuit to induce the corresponding electrical displacement current. When they came into contact again, the EDL was rebuilt, and the ions in the diffuse layer migrated in the opposite direction under the triboelectric‐induced polarization by the Stern layer, inducing a reverse electrical displacement current in the external circuit. Repeated cycling resulted in an AC output, with the diffuse layer acting as the primary region of ion rearrangement. Its open‐circuit voltage (*V*_OC_) reached ≈52 V, the short‐circuit current (*I*_SC_) density approached 2.45 mA m^−2^, and the peak power (*P*_R_) density of nearly 0.13 W m^−2^ (Figure 4c). The device could successfully power 60 LEDs, establishing a foundation for SL‐TENG functionality in water‐based environments. Moreover, the dependence of output on wave frequency and liquid composition suggested the potential for environmental sensing applications.

To extend SL‐TENG applications from bulk water motion to individual droplets, a refined structure was introduced by Lin et al. in 2014^[^ ^88^ ^]^ (Figure 4d). In this version, a PTFE film with hierarchical micro/nanostructures was employed to create a superhydrophobic surface, minimizing wetting hysteresis and enabling rapid droplet detachment. The PTFE was deposited onto a Cu‐coated PMMA substrate, forming a single‐electrode energy harvesting unit. The operating mechanism involved two sequential processes. First, as the water droplets continuously hit the PTFE surface after free fall, charge transfer occurred, prompting the negatively charged Stern layer to gradually form on the solid surface. Second, as the subsequent water droplets gradually approached and left the PTFE surface, the dynamic changes in triboelectric‐induced polarization by the Stern layer continuously drove the reciprocating migration of ions in the diffuse layer, thereby driving the continuous reciprocating electron transfer between the Cu electrode and the ground. As the droplet fell from a higher height, the impact force on the interface increased, resulting in more charge transfer (Figure 4e). After the droplet fell to a height of 20 cm, the performance gradually stabilized. The *V*_OC_ and *I*_SC_ were moderate at 9.3 V and 17 μA, respectively (Figure 4f), and the system exhibited stable AC output during repetitive droplet impact. Additionally, the superhydrophobic design ensured long‐term operability even under high humidity and elevated temperatures. This work expanded the SL‐TENG platform toward droplet‐scale energy harvesting, offering new opportunities for raindrop‐powered electronics and autonomous environmental sensors. Although early SL‐TENGs offered a promising platform for harvesting energy from dynamic interfaces, their performance was often constrained by limited instantaneous output and suboptimal interfacial charge collection. A pivotal advancement emerged in 2020 with the introduction of a droplet‐based electricity generator (DEG) proposed by Xu et al.^[^ ^131^ ^]^ (Figure 4g), which exhibited a substantial performance enhancement attributed to a newly identified “bulk effect.” In this architecture, water droplets impinging upon a negatively charged PTFE surface initially contacted an overlying aluminum (Al) electrode, forming a vertical charge‐transfer junction. The triboelectric‐induced polarization of the charged PTFE surface induced the ions in the diffuse layer to migrate rapidly toward the Al electrode. This enabled rapid and large‐scale charge redistribution between the Al and the indium tin oxide electrode via electrostatic induction (Figure 4h), rather than relying on localized interfacial electron exchange. As the droplet continued to move slowly on the Al electrode, a slower charge reflow was induced due to the redistribution of the induced charges, similar to the behavior of a dynamic capacitive switch. This new mechanism of “bulk effect” resembling capacitive switching resulted in a dramatic increase in the output, where the *V*_OC_ reached 143.5 V and *I*_SC_ exceeded 270 μA per droplet. The corresponding instantaneous results far exceeded those of single‐electrode configurations without the Al electrode. Notably, the DEG also demonstrated excellent operational stability under varying environmental conditions, including high humidity, and exhibited compatibility with a broad range of droplet sizes and electrode materials, indicating the generalizability of the mechanism across system designs.

These three generations of SL‐TENGs illustrate a clear evolution from simple contact‐separation systems to complex, dynamically coupled electrostatic architectures. Early devices relied on interfacial charge accumulation and diffuse layer modulation, and the most recent advances introduced internal circuit dynamics based on capacitive bridging and rapid field redistribution. Together, these developments not only improved performance metrics by several orders of magnitude but also diversified the operational environments of SL‐TENGs, from waves and rain to tap water, microdroplets, and dynamic flow fields. The trajectory of SL‐TENG innovation demonstrates that systematic control over surface topography, interfacial chemistry, and spatially coupled electrode design can collectively enable programmable and scalable energy harvesting from ubiquitous water sources.

#### 3.1.2 CS‐TINGs

**Figure 5:**
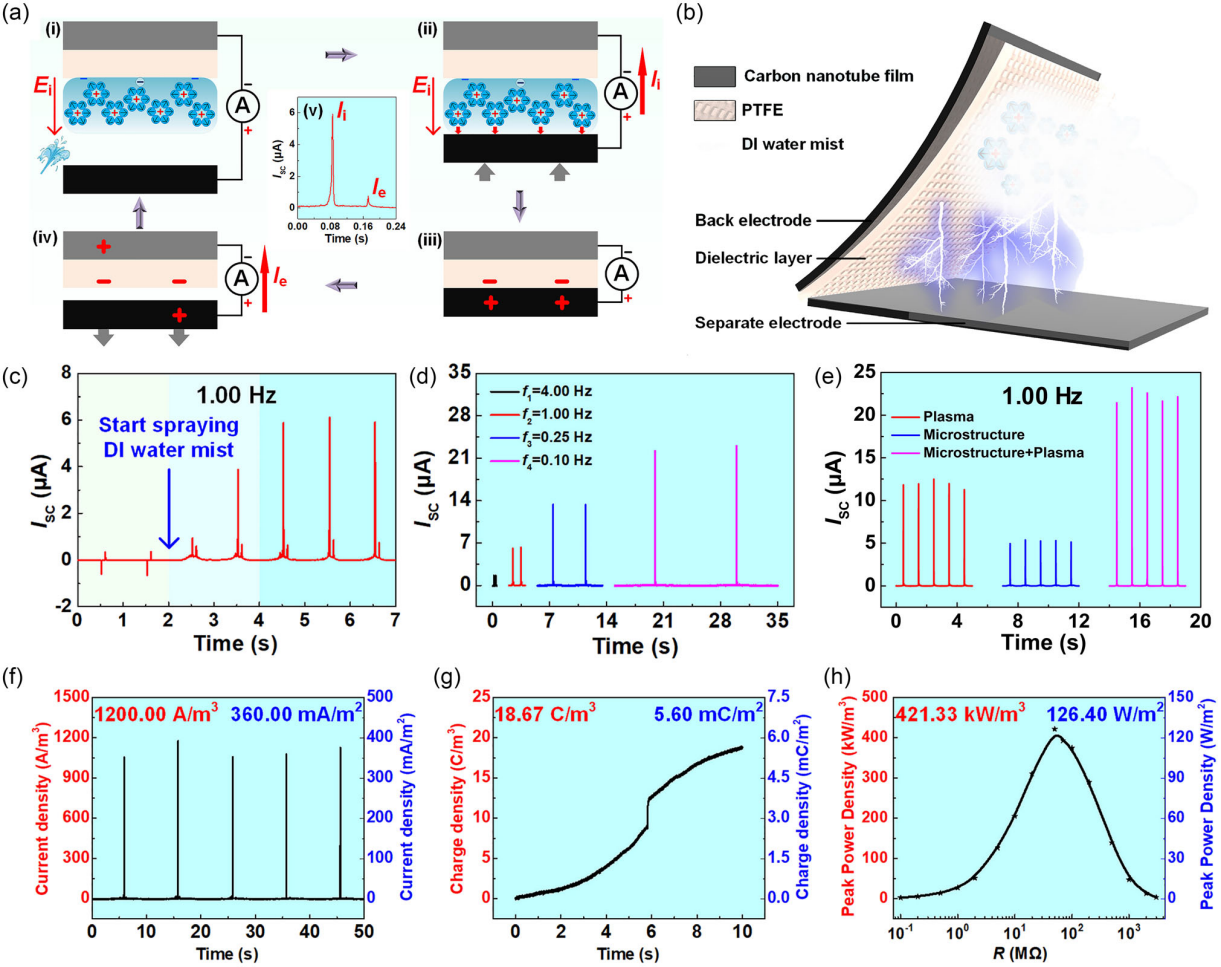
The CS‐TING was developed by Wei et al. in 2023, which incorporated a self‐replenishing ionic strategy to dynamically modulate interfacial charge states. Reproduced with permission.^[^ ^80^ ^]^ Copyright 2023, Elsevier Inc. a) Demonstration of the operating mechanism. b) Schematic diagram of the device structure. c) Experiments showed that the ionic current generated from the charged‐mist‐augmented diffuse layer was found to be synergistically aligned with the electronic displacement current, yielding enhanced DC output. d–e) To further enhance performance, two targeted strategies were adopted: reducing operational frequency to prolong charge enrichment time, and surface engineering of the PTFE layer to enhance hydrophilicity. f–h) Characterization of the output performance of the device.

While SL‐TENGs effectively harvest energy through the transient migration of mobile ions in the diffuse layer of the EDL, their energy conversion efficiency is inherently constrained by the limited density of free charge carriers within that region. Moreover, once charge transfer occurs, the residual charge on the dielectric surface might tend to decay due to environmental influences such as temperature and humidity, leading to unstable or diminishing output. To overcome these fundamental limitations, as a new class of devices, charge‐supplementary triboiontronics nanogenerators (CS‐TING) was developed by Wei et al. in 2023,^[^ ^80^ ^]^ incorporating a self‐replenishing ionic strategy to dynamically modulate interfacial charge states (**Figure** **5**a). This approach introduced precharged water mist, generated via solid–liquid contact electrification, as a mobile ionic reservoir that was continuously deposited onto the dielectric surface. The positive ions in the water mist could enrich the diffuse layer and establish an ion concentration gradient, thereby inducing an ionic current. It was coupled with the electrical displacement current induced during the contact‐separation cycles to synchronize DC ionic‐electronic coupling output, effectively shifting the working principle from purely electrostatic induction toward a hybrid field‐assisted charge transport mechanism. Structurally, the CS‐TING device consisted of a PTFE dielectric layer interfaced with carbon nanotube (CNT) films serving as both back and movable electrodes (Figure 5b). This configuration not only ensures a large surface area for ionic accumulation but also enhances environmental stability, especially under high humidity conditions where conventional metal electrodes are susceptible to corrosion. Experiments showed that the ionic current generated from the charged‐mist‐augmented diffuse layer was found to be synergistically aligned with the electronic displacement current, yielding enhanced DC output (Figure 5c). Control experiments, such as replacing water mist with concentrated electrolyte solutions, blocking ionic transfer with insulating oil layers, or operating in dry conditions, confirmed the critical role of the pre‐charged mist and the dynamic modulation of the diffuse layer in enabling the observed DC behavior. These results establish CS‐TINGs as a fundamentally distinct operational regime within triboiontronics.

To further enhance performance, two targeted strategies were adopted (Figure 5d,e): reducing operational frequency to prolong charge enrichment time, allowing more mist to accumulate and thereby increasing ionic charge storage, and surface engineering of the PTFE layer to enhance hydrophilicity through micro‐engraving and plasma treatment. These modifications significantly improved the interfacial ion‐adsorption capacity, reduced the contact angle from 100° to below 20°, and resulted in a remarkable increase in output metrics. Under optimized conditions (0.1 Hz operating frequency with dual‐treated PTFE), the CS‐TING achieved an *I*_SC_ of 36.0 μA, *Q*_SC_ of 560.0 nC, *V*_OC_ of 1750 V, and *P*_R_ of 12.64 mW (Figure 5f–h). These values translate to a current density of 360 mA m^−2^ and a power density of 126.4 W m^−2^ (421.33 kW m^−3^), orders of magnitude higher than conventional SL‐TENGs and surpassing many state‐of‐the‐art DC‐TENG platforms that rely on mechanical rectification, semiconductor junctions, or dielectric breakdown. Beyond performance, the CS‐TING also demonstrated superior application versatility. It enabled direct charging of capacitors and lighting of LED arrays without auxiliary circuitry; increased the effective internal capacitance of the system through the formation of a dynamic, water‐film‐mediated EDL; and avoided performance degradation under fluctuating environmental conditions. These characteristics suggest that CS‐TINGs could not only resolve several key bottlenecks faced by traditional triboelectric systems, but also offer a robust, scalable, and material‐generalizable platform for high‐efficiency energy harvesting. Furthermore, for CS‐TING devices, the voltage output arises from the ionic supplementation of the EDL diffuse layer by charged microdroplets, under ambient mechanical modulation. The observed macroscopic output voltages range from hundreds to thousands of volts, far exceeding the typical millivolt‐scale streaming potentials generated by pressure‐driven flow in nanochannels or capillaries. In addition, the control experiment was conducted in which higher‐concentration LiCl solutions were sprayed onto the device using a humidity generator. Due to strong EDL screening effects from the high ionic strength, the droplets became uncharged, and CS‐TING output was effectively suppressed. This confirms that the voltage output originates from the dynamic charge supplementation to the EDL, not from redox reactions, since high‐concentration electrolytes typically enhance rather than inhibit redox activity.

Overall, CS‐TINGs represented a significant evolution from conventional SL‐TENGs by addressing a key limitation: the static and insufficient charge density within the diffuse layer. By introducing a source‐free ionic charge supplementation mechanism, CS‐TINGs unlocked a new paradigm in triboiontronics, one that allowed for controllable and regenerative ionic‐electronic coupling at dielectric‐liquid interfaces. This innovation could not only improve energy output and efficiency but also eliminate reliance on external rectification circuits, expanding the functional range of TENGs into high‐power and high‐stability domains. As a bridge between electrostatics, ionics, and mechanical energy harvesting, CS‐TINGs paved the way for next‐generation self‐powered iontronics, intelligent sensing interfaces, and distributed liquid‐interfacing power modules.

### 3.2 Energy Technologies Based on the Dynamic Regulation of the Entire EDL

While SL‐TENGs and CS‐TINGs have demonstrated effective water‐based energy harvesting by dynamically regulating the ionic charge distribution in the diffuse layer of the EDL, their output capacity remains constrained by the limited volume and carrier density within this outermost region. To further enhance the energy conversion efficiency, strategies have been proposed to modulate the entire EDL, encompassing both the compact Stern layer and the extended diffuse layer. By simultaneously engaging all interfacial substructures in the charge transport process, a higher density of transferred charge and more sustained output can be achieved. In this context, two representative approaches have emerged. On the one hand, hydrovoltaic nanogenerators utilize the directional movement of liquid‐solid interfaces to induce asymmetric ion migration along a moving EDL boundary,^[^ ^132^, ^133^, ^134^, ^135^, ^136^, ^137^ ^]^ typically yielding low‐amplitude constant signals. On the other hand, TINGs based on asymmetric EDLs can generate pulsed DC outputs with significantly enhanced current amplitudes, enabled by persistent interfacial asymmetry and stable ion concentration gradients. These systems exemplify the potential of entire‐EDL modulation for higher‐performance mechanical‐to‐electrical energy conversion.

#### 3.2.1 Hydrovoltaic Nanogenerators

**Figure 6:**
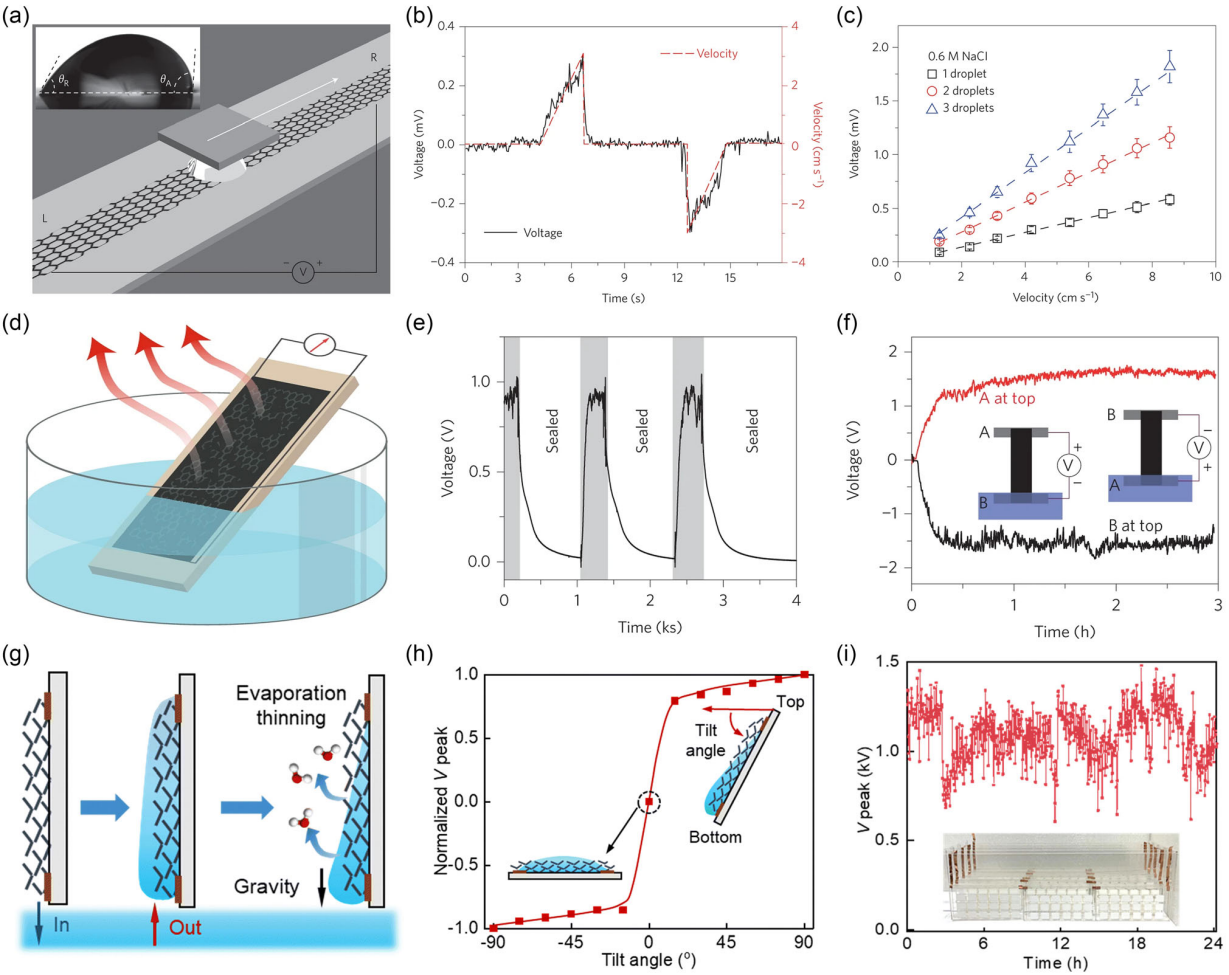
A series of hydrovoltaic nanogenerators and their output characteristics. a) The earliest hydrovoltaic nanogenerator was proposed by Yin et al. in 2024. Reproduced with permission.^[^ ^94^ ^]^ Copyright 2014, Springer Nature Limited. b–c) The voltage magnitude was scaled linearly with both droplet velocity and number. d) The hydrovoltaic nanogenerator based on carbon‐based porous films was proposed by Xue et al. in 2017. Reproduced with permission.^[^ ^84^, ^93^ ^]^ Copyright 2018, The Author(s), Copyright 2017, Springer Nature Limited. e) Experimental control through humidity, temperature, and wind confirmed the dominant role of evaporation in driving interfacial ionic movement. f) The generated voltage was scaled with both environmental parameters and device orientation. g) Deng et al. developed a more efficient hydrovoltaic nanogenerator in 2023. Copyright 2023, RSC Publishing.^[^ ^135^ ^]^ Copyright 2023, RSC Publishing. h) EDL boundary movement was simultaneously governed by streaming and evaporation‐induced potentials. i) Integration of multiple devices in series generated a *V*_OC_ exceeding 1200 V.

Hydrovoltaic nanogenerators represent a class of devices that convert interfacial water dynamics, such as droplet movement, evaporation, or capillary flow, into electrical energy through the movement of the EDL boundary. The earliest hydrovoltaic nanogenerator was proposed by Yin et al. in 2024,^[^ ^94^ ^]^ involving millivolt‐level voltage signals generated by translating ionic solution droplets across monolayer graphene on the PET substrate (**Figure** **6**a). This process activated a mobile EDL at the droplet‐graphene/PET interface. As the EDL boundary moved with the droplet, a dynamic imbalance in ion charge density at the leading and trailing edges resulted in a voltage whose magnitude scaled linearly with both droplet velocity and number (Figure 6b,c). The phenomenon, termed “drawing potential,” was highly sensitive to ionic species, with Li^+^, Na^+^, and K^+^ generating positive voltages and HCl producing reverse polarity due to surface protonation. Multilayer graphene samples exhibited reduced output due to reduced direct contact between the liquid and the dielectric, confirming the importance of the dielectric substrate in constructing a stable EDL to ensure output performance. Moreover, experiments have shown that the output polarity of hydrovoltaic nanogenerators is consistently aligned with the droplet motion direction, consistent with EDL boundary displacement but inconsistent with faradaic (redox) processes, which would not exhibit directional dependence in the absence of redox asymmetry. These results firmly support EDL boundary migration as the dominant mechanism, rather than streaming potential or electrochemical reactions. Device simplicity, directional sensitivity, and reproducibility under ambient conditions position this mechanism as a bridge between electrokinetics and 2D‐material electronics, opening pathways toward graphene‐based sensors and self‐powered interfaces.

In addition to the drag of the droplet, the hydrovoltaic nanogenerator based on carbon‐based porous films was proposed by Xue et al. in 2017, which achieved spontaneous voltage generation by natural water evaporation^[^ ^84^, ^93^ ^]^ (Figure 6d). In this system, a flame‐deposited nanostructured carbon black (CB) film on the PET functionalized to achieve high hydrophilicity could enable capillary‐driven water infiltration and directional evaporation. The movement of water molecules through the porous matrix, coupled with their interaction with oxygen‐containing surface groups, led to dynamic EDL boundary movement and charge redistribution, thereby forming the streaming potential‐like signals. Therefore, the direction of the current generated by this hydrovoltaic nanogenerator depended on the flow direction of the liquid. Sustained *V*_OC_ up to 1 V and stable *I*_SC_ (≈150 nA) were achieved under ambient conditions for several days. Spatial voltage mapping using multielectrode arrays further confirmed that electricity was exclusively generated in regions where water evaporation occurred, with no measurable output in submerged or completely dry regions. Experimental control through humidity, temperature, and wind confirmed the dominant role of evaporation in driving interfacial ionic movement (Figure 6e). Density functional theory (DFT) calculations revealed that specific functional groups (e.g., C‐*O*‐C) on disordered graphene domains facilitated the formation of interfacial dipoles and electron depletion layers, thus enhancing charge separation. The generated voltage was scaled with both environmental parameters and device orientation (Figure 6f). Series/parallel integration of multiple devices enabled practical applications such as powering display units. These findings established evaporation‐driven electrokinetic effects as a viable mechanism for ambient‐condition energy harvesting. Based on this evaporation mechanism, Deng et al. developed a more efficient hydrovoltaic nanogenerator by refining both the material platform and the spatial configuration of the active interface in 2023^[^ ^135^ ^]^ (Figure 6g). In this device, a porous semiconducting bismuth oxyhalide nanoplate (BiOCl) film was employed as the active material, leveraging its high surface zeta potential and aqueous stability. A novel “water‐film mode” was introduced, wherein the BiOCl surface was coated with a thin water layer that progressively thinned and receded under the influence of gravity and evaporation. This created a semi‐wet capillary front as a dynamic region rich in gas–liquid–solid interfaces, where charge migration induced by EDL boundary movement was simultaneously governed by streaming and evaporation‐induced potentials (Figure 6h). Unlike conventional immersion‐based devices, this configuration avoided bulk water short‐circuiting and enabled broader interfacial activity. Infrared spectroscopy and section‐resolved voltage profiling verified that voltage generation was spatially confined to the advancing capillary front, with the voltage increasing as the wet‐dry interface migrated across the device. Integration of multiple devices in series generated a *V*_OC_ exceeding 1200 V (Figure 6i), sufficient to drive atmospheric helium ionization, demonstrating the system's capacity for higher‐voltage applications. Theoretical modeling further suggested that water molecule desorption led to net hole accumulation in the BiOCl film, providing the physical basis for the evaporative potential. This system highlighted the importance of engineering interfacial phase boundaries and nanoscale hydration gradients as a strategy for amplifying hydrovoltaic output, while also showcasing the potential for scalable integration.

In summary, hydrovoltaic nanogenerators have emerged as a promising class of solid–liquid interface devices capable of converting ambient mechanical and thermal energy into electricity through dynamic regulation of the EDL. The underlying mechanisms, whether based on mobile pseudocapacitors driven by droplet motion, evaporation‐induced streaming potentials, or hybrid effects at semi‐wet capillary fronts, all converge on the principle of ion redistribution across nanoscale interfacial gradients, resulting in electric potential generation without external bias. Despite their diversity, these systems are unified by their reliance on EDL boundary movement across spatiotemporally evolving solid–liquid interfaces.

#### 3.2.2 Triboiontronics Nanogenerators Based on Asymmetric EDLs

While hydrovoltaic nanogenerators have demonstrated notable advances in ambient‐condition power generation, the achievable current density and power output remain limited by the extent of dynamic EDL modulation. In this context, asymmetrically structured EDLs offer a pathway toward enhanced charge separation and unidirectional ionic‐electronic coupling. By designing built‐in interfacial asymmetry at the molecular or architectural level, TINGs based on asymmetric EDLs could enable directional ionic currents and programmable charge flow, laying the foundation for efficient mechanical‐to‐electrical energy conversion and ionic circuit control.

**Figure 7:**
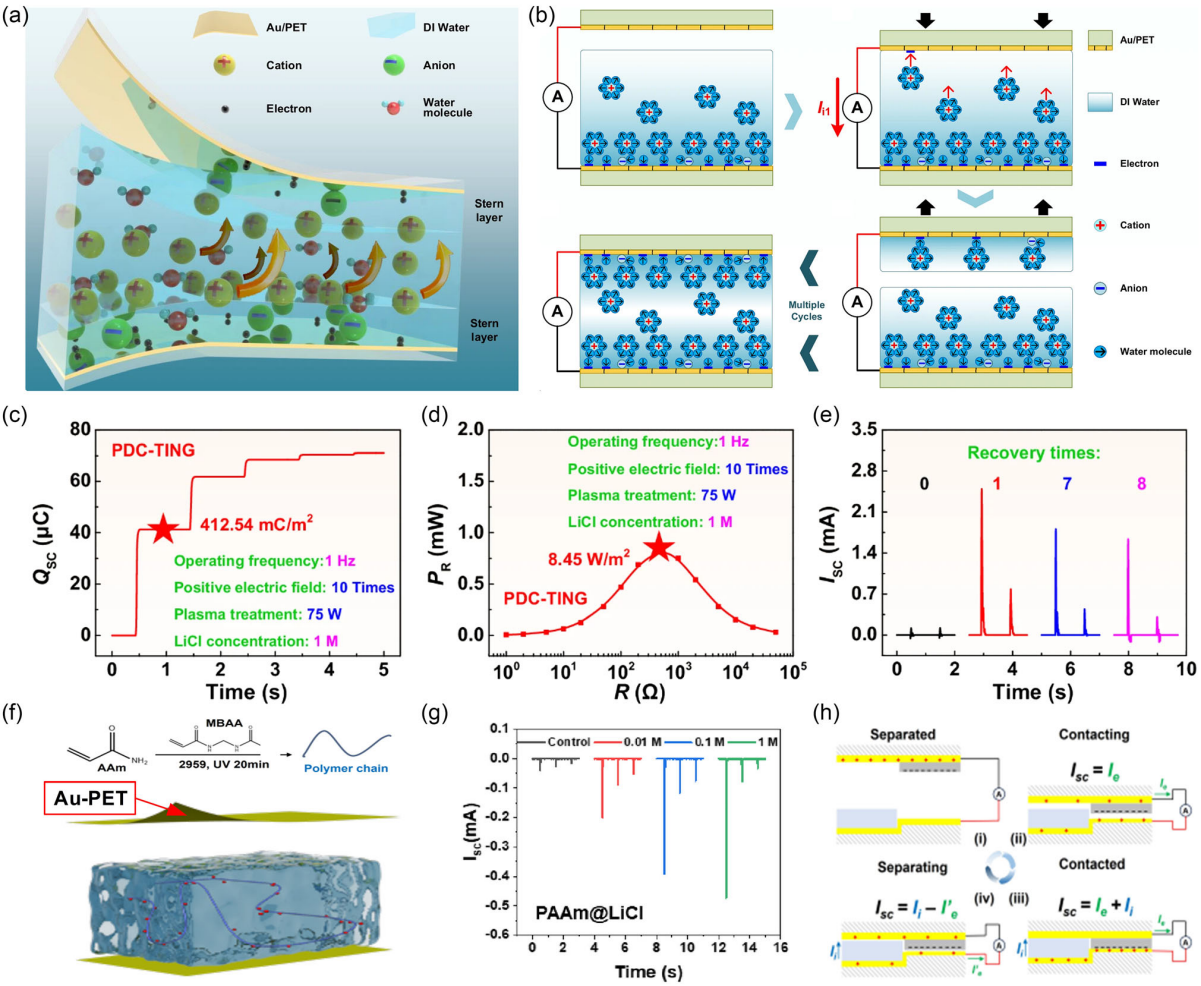
A series of TINGs based on asymmetric EDLs and their output characteristics. a) The PDC‐TING was developed by Li et al. in 2024. Reproduced with permission.^[^ ^98^ ^]^ Copyright 2024, The Author(s). b) The operating mechanism of the PDC‐TING. c) It could achieve a *Q*_SC_ density of over 412 mC/m^2^. d) The PDC‐TING could realize a *P*_R_ density of 8.45 W/m^2^. e) The asymmetry of the EDLs could be restored by the electrochemical recovery method. f) The TING proposed by Ouyang et al. in 2024 could leverage the dynamic formation of asymmetric EDLs to realize ionic current rectification. Reproduced with permission.^[^ ^138^ ^]^ Copyright 2024, American Chemical Society. g) The ionic conductivity and output current were further enhanced by increasing the ion concentration. h) Integrating this hydrogel‐based TING with a conventional TENG allowed the ionic current to rectify the displacement current.

One representative strategy involves regulating EDL asymmetry by engineering the solid‐liquid interfacial properties via surface metallization. Based on this, physically driven direct‐current TING (PDC‐TING) was developed by Li et al. in 2024^[^ ^98^ ^]^ (**Figure** **7**a). In this device, an ultrathin metal layer such as gold (Au) was sputtered onto the PET substrate to serve as a charge‐collecting electrode. By controlling the metal coverage and introducing microscale cracks, selective exposure of the dielectric to the liquid phase was achieved. This resulted in the formation of a distinct asymmetric EDL between the top and bottom surfaces during the contact‐separation process (Figure 7b). The resulting larger ion concentration gradient drove efficient ion migration and induced electron transfer in the external circuit, resulting in a DC output with a higher transferred charge density. Notably, this configuration could achieve a *Q*_SC_ density of over 412 mC m^−2^ (Figure 7c), figures substantially superior to conventional water‐based energy harvesting devices based on EDL dynamic regulation. In addition, the PDC‐TING could realize a *P*_R_ density of 8.45 W m^−2^ (Figure 7d). To further improve operational stability, after the asymmetry of the EDLs was destroyed, the asymmetry could be restored by the electrochemical recovery method (Figure 7e), thereby ensuring the device could be reused. Importantly, these asymmetric EDLs could be finely modulated through external variables such as substrate wettability (e.g., via plasma treatment), electrostatic prepolarization, and the ion concentration of the contacting liquid, each of which adjusts the local charge density and ion flux across the EDL.

Complementing this approach, another TING design leveraged the dynamic formation of asymmetric EDLs at hydrogel‐ultrathin metal/dielectric interfaces to realize ionic current rectification^[^ ^138^ ^]^ (Figure 7f), which was proposed by Ouyang et al. in 2024. In this system, polyacrylamide (PAAm)‐based ionogels, either salt‐free or doped with LiCl or Li[TFSI], were cyclically contacted with Au/PET film. The inherent water content within the hydrogel facilitated asymmetric EDL formation through hydration‐driven ion accumulation on the Au/PET surface. The larger ion concentration gradient between the asymmetric EDLs induced unidirectional ion migration, resulting in a stable DC signal. In particular, under low‐humidity conditions, dehydration of the hydrogel slowed EDL equilibration and prolonged the DC output duration, reaching up to 10 min in salt‐free configurations. The ionic conductivity and output current were further enhanced by increasing the ion concentration, achieving *I*_SC_ values of ≈0.6 mA and sustaining a *V*_OC_ of ≈0.8 V when using hydrogels with 1 M LiCl (Figure 7g). Moreover, integrating this hydrogel‐based TING with a conventional TENG allowed the ionic current to rectify the displacement current (Figure 7h), offering a platform for self‐regulated hybrid ionic‐electronic energy systems.

**Figure 8:**
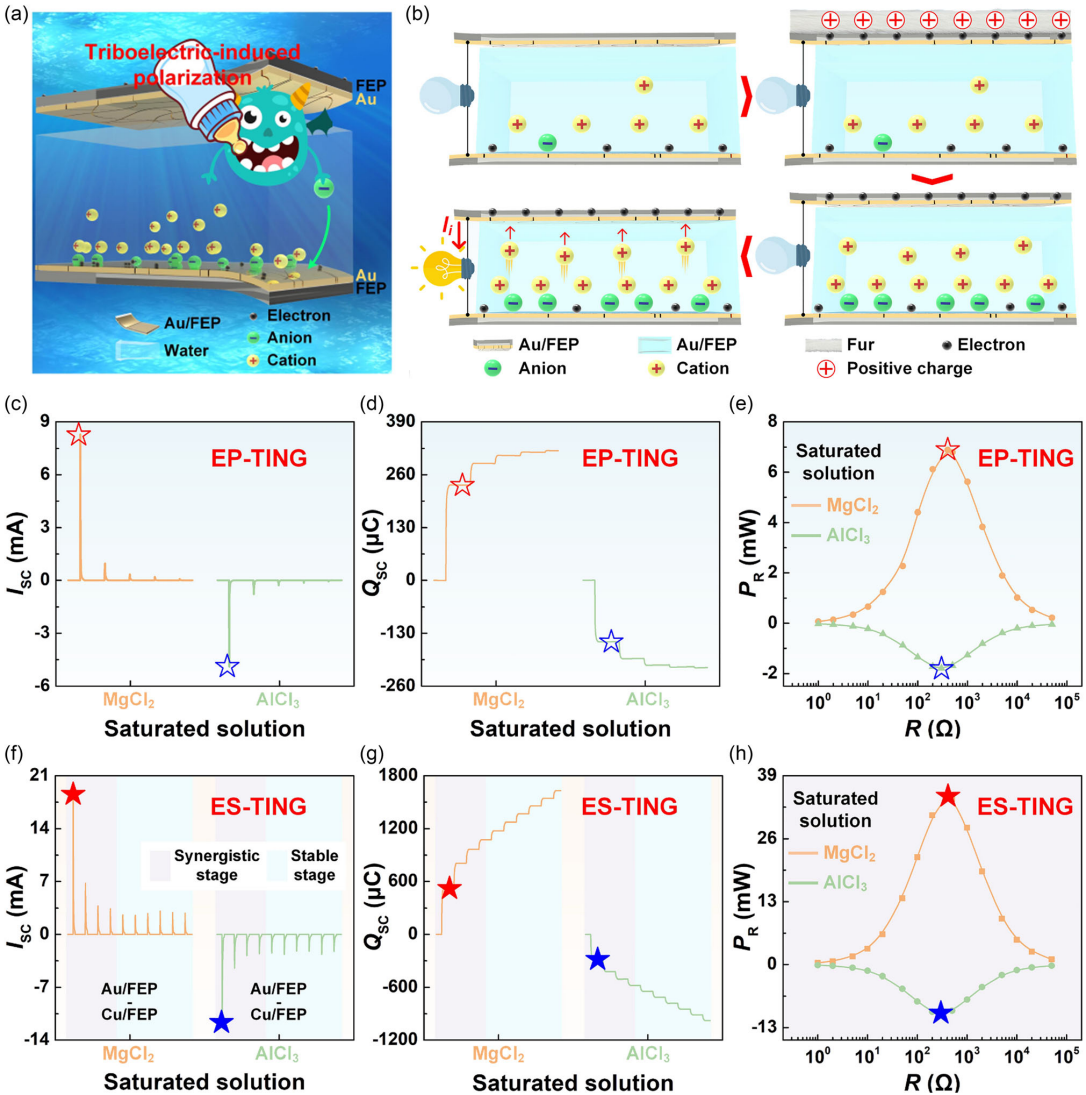
To further enhance the output performance of TINGs, triboelectric‐induced polarization can be harnessed to actively modulate asymmetric EDLs, thereby amplifying the ion concentration gradient between two interfacial EDLs. a) An EP‐TING was developed by Li et al. in 2025. Reproduced with permission.^[^ ^99^ ^]^ Copyright 2025, Elsevier Inc. b) The operating mechanism of the device. c–e) Output characteristics of the EP‐TING. f–h) Output characteristics of the ES‐TING.

To further enhance the output performance of TINGs, recent research has demonstrated that triboelectric‐induced polarization can be harnessed to actively modulate asymmetric EDLs, thereby amplifying the ion concentration gradient between two interfacial EDLs. This concept, inspired by the function of Maxwell's demon, enables remote and noncontact regulation of interfacial charge distribution via electrostatic fields generated through solid–solid contact electrification. In this context, an enhanced physical adsorption TING (EP‐TING) was developed by Li et al. in 2025^[^ ^99^ ^]^ (**Figure** **8**a), which could realize effective energy flow. In this system, a triboiontronics Maxwell's demon was constructed by applying triboelectric‐induced polarization across hybrid metal/dielectric films. Specifically, a dielectric substrate (e.g., FEP) coated with an ultrathin Au layer was used to form the EP‐TING. When the FEP layer was pre‐charged through contact electrification with electropositive materials such as fur, a stronger negative surface potential was induced, which remotely drove anions toward the bottom EDL interface and enhanced cation separation, leading to an intensified ion concentration gradient (Figure 8b). Upon subsequent contact with water, the top Au/FEP layer completed the EDL pair, generating a highly asymmetric configuration that enabled more efficient ion migration and boosted electron transfer in the external circuit. This remote polarization strategy led to a notable increase in transferred charge density, thereby effectively improving the output performance. This optimization resulted in an *I*_SC_ density of 83 A/m^2^, *Q*_SC_ density of 2347.4 mC m^−2^, and *P*_R_ density of 68.9 W m^−2^ in the EP‐TING with saturated MgCl_2_ as the electrolyte, substantially higher than that of conventional TING designs (Figure 8c‐e).

Furthermore, the synergistic integration of redox reactions gave rise to the enhanced synergy strategy TING (ES‐TING), wherein redox‐active metals such as Cu were introduced to dynamically couple ion migration with faradaic processes. In the case of Cu/FEP film as the bottom layer and saturated MgCl_2_ as the electrolyte, the ES‐TING achieved an exceptional *Q*_SC_ density of 5237.51 mC m^−2^, with an *I*_SC_ density of 186.2 A m^−2^ and a *P*_R_ density of 346.7 W m^−2^ (Figure 8f–h). Importantly, this triboiontronics architecture also enabled programmable control over carrier polarity and current direction through selective ion species (e.g., Li^+^, Mg^2+^, Al^3+^, or H^+^) and electrostatic field polarity, allowing the device to serve as a logical ionic‐electronic rectifier. At the material level, both the dielectric's electronegativity and the metal layer thickness were found to critically influence EDL behavior. Higher electronegativity substrates enhanced CE‐driven charge accumulation, while optimal metal coverage (e.g., 10 min sputtered Au) balanced interfacial conductivity and ion access via microscopic cracks. Excessive sputtering reduced contact electrification efficiency by fully screening the dielectric, thus weakening EDL asymmetry and device output. Moreover, adjusting the surface hydrophilicity of the Au/FEP layer through plasma treatment further modulated the bottom EDL density, enabling fine control over performance. Under optimized conditions, the EP‐TING and ES‐TING exhibited robust reusability and longer‐term output stability through electrochemical recovery cycles and material design strategies. This triboiontronics Maxwell's demon via triboelectric‐induced polarization could not only improve energy harvesting performance but also offer a framework for regulating energy‐information conversion at the ionic‐electronic interface. Its relevance extended beyond power generation to bionic neuromorphic systems and integrated logic‐controlled platforms, thereby opening new avenues for energy‐autonomous, multifunctional systems in the post‐Moore era.

The development of TINGs based on asymmetric EDLs presented a powerful strategy to bridge ionic and electronic transport through dynamic solid‐liquid interface engineering. By exploiting interfacial asymmetry, either through structural design (e.g., Au/PET bilayers), material modulation (e.g., hydrogel iontronics), or external triboelectric‐induced polarization (e.g., triboiontronics Maxwell's demon), these systems achieved enhanced ion concentration gradients, controllable charge carrier polarity, and efficient energy‐information transduction. Compared to conventional water‐based energy harvesting devices based on EDL dynamic regulation, TINGs demonstrated substantially improved output metrics, including *Q*_SC_ density exceeding 5,000 mC m^−2^, along with superior flexibility, stability, and compatibility with self‐powered logic or neuromorphic circuits. More importantly, the capacity to dynamically program interfacial charge behavior via contact electrification or work function engineering enabled TINGs to not only harvest energy but also actively participate in ionic signal routing and processing. These advances collectively established triboiontronics systems as a foundational platform for next‐generation soft electronics, intelligent sensing, and autonomous human‐machine interfaces.

In summary, the dynamic regulation of the EDL provided a powerful and unifying framework for the development of next‐generation energy harvesting and conversion systems. By moving beyond static models and instead leveraging the EDL as a tunable, stimulus‐responsive interfacial structure, diverse mechanisms, ranging from electrostatic coupling in SL‐TENGs to directional ionic transport in hydrovoltaic nanogenerators and TINGs, have demonstrated enhanced control over charge separation, ion selectivity, and output polarity. These systems illustrated how modulation of different EDL subdomains, whether the diffuse layer or the entire interfacial architecture, could be tailored to generate either transient or continuous electrical outputs with application‐specific performance metrics. As the field progresses, integrating dynamic EDL engineering with advanced materials and device architectures promises not only to improve energy conversion efficiency but also to enable adaptive, self‐powered platforms for sensing, communication, and neuromorphic computation. This convergence of interfacial science and triboiontronics control underscores the transformative potential of EDL‐centric approaches in reshaping the landscape of soft energy and information technologies.

## 4 Information Scavenging and Modulation Based on Dynamic Regulation of the EDL

While the dynamic regulation of the EDL has shown great promise in enhancing energy harvesting efficiency through tailored ionic‐electronic coupling, its implications extend far beyond energy conversion alone. The same interfacial mechanisms that enable efficient charge transduction can also be harnessed for information acquisition, signal modulation, and neuromorphic computation. By exploiting the sensitivity of the EDL to external mechanical, chemical, and electrostatic stimuli, researchers have begun to develop EDL‐based platforms capable of transducing subtle interfacial perturbations into interpretable electrical signals. More significantly, when the regulation extends from the diffuse layer to the entire EDL architecture, complex ionic behaviors, such as memory, logic, and communication, can be orchestrated in a fully self‐powered manner. This opens a new frontier where energy and information are not only co‐localized at soft interfaces, but co‐evolved through unified EDL dynamics, thereby laying the foundation for information scavenging and modulation.^[^ ^139^, ^140^, ^141^, ^142^, ^143^, ^144^, ^145^, ^146^, ^147^, ^148^ ^]^

### 4.1 Information Scavenging Based on the Dynamic Regulation of the Diffuse Layer

The dynamic behavior of the diffuse layer offers a sensitive mechanism for monitoring interfacial charge transfer processes, enabling the development of self‐powered, EDL‐based interface probes. Recent studies have demonstrated how the regulation of the diffuse layer, particularly in solid–liquid contact electrification systems, can be harnessed to achieve high‐resolution readouts of environmental or phase‐state changes.

**Figure 9:**
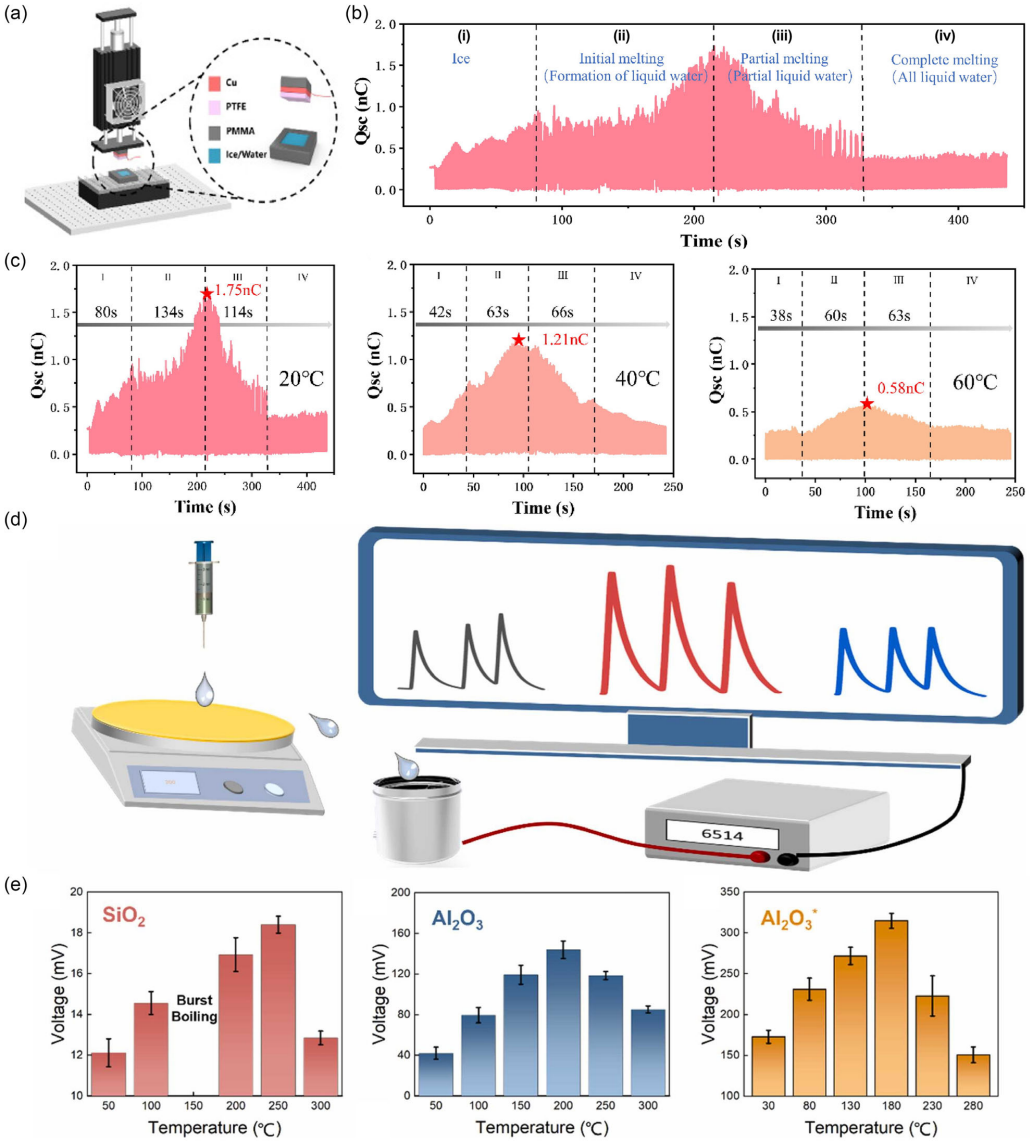
Dynamic regulation of the diffuse layer in EDLs enables self‐powered interfacial probing. a) SL‐TENG was used to investigate interfacial charge behavior during the ice‐to‐water phase transition by Wei et al. in 2024. Reproduced with permission.^[^ ^100^ ^]^ Copyright 2024, Elsevier Ltd. b) The melting process reveals how the shift from solid–solid to solid‐liquid contact influences charge dynamics. c) Temperature‐controlled tests confirm that gradual EDL formation modulates charge screening. d) SL‐TENG was applied to study the Leidenfrost effect and charge transfer under thermally dynamic conditions by Li et al. in 2025. Reproduced with permission.^[^ ^149^ ^]^ Copyright 2024, Elsevier Ltd. e) The observed non‐monotonic charge response reflects dynamic changes in droplet–surface contact and EDL behavior.

In one representative study by Wei et al. in 2024,^[^ ^100^ ^]^ researchers utilized a single‐electrode SL‐TENG based on dynamic regulation of the diffuse layer as a probe to investigate the contact electrification behavior during the phase transition of ice to water (**Figure** **9**a). The melting process of the ice provided a unique platform to analyze how the transformation from solid–solid to solid–liquid contact affects interfacial charge dynamics (Figure 9b). Initially, electron‐dominated contact electrification at the solid‐solid interface contributed to low charge transfer. As melting progressed, microdroplets smoothed the ice surface and increased the contact area, resulting in a sixfold enhancement in charge output. However, once sufficient liquid water accumulated, an EDL formed at the solid–liquid interface. This EDL exerted a screening effect on the net surface charges, effectively impeding further charge transfer. The evolution of the transferred charge, from initial amplification due to increased contact area, to eventual decline driven by EDL formation, reflected the temporal dynamics of diffuse layer evolution. The temperature‐controlled experiments further validated that the emergence of free ions and the progressive establishment of the EDL were key in modulating charge screening (Figure 9c), especially under varying hydrophilicity and ion concentration. This study not only elucidated the role of EDL in charge attenuation but also established SL‐TENG as a sensitive probe to monitor subtle physicochemical transitions at interfaces, with implications for environmental sensing and polar expedition instrumentation.

Complementing this, another investigation proposed by Li et al. in 2025 employed an SL‐TENG to probe the Leidenfrost effect,^[^ ^149^ ^]^ where a vapor layer forms between a droplet and a hot surface above a critical temperature (Leidenfrost point), to explore charge transfer under thermally dynamic solid–liquid interactions (Figure 9d). Triboelectric voltage signals from sliding droplets were measured across a wide temperature range on different substrates. Remarkably, the output voltage peaked near the Leidenfrost point, indicating a maximal interfacial charge transfer just before complete vapor insulation. This nonmonotonic behavior, charge increasing with temperature up to the Leidenfrost point and decreasing thereafter, was attributed to the dynamic regulation of droplet‐surface contact states, which directly affected EDL formation and charge exchange (Figure 9e). The introduction of salts and ionic liquids showed that variations in ion type and concentration could shift the Leidenfrost point and modulate contact electrification intensity, validating the robustness of SL‐TENG‐based Leidenfrost point detection. Furthermore, simulations demonstrated how droplet levitation altered diffuse layer characteristics by minimizing direct interfacial interactions, emphasizing that both mechanical and thermal boundary conditions regulate diffuse layer‐mediated charge dynamics. This work not only revealed the intimate coupling between contact electrification and thermal interfacial phenomena but also positioned SL‐TENGs as real‐time probes for monitoring heat transfer regimes, offering applications in high‐temperature energy devices, surface engineering, and smart thermal management.

Together, these two studies underscore the critical role of diffuse layer dynamics in mediating charge exchange at solid–liquid interfaces. Whether through temperature‐induced phase transitions or thermally driven wetting changes, the modulation of diffuse layer composition and spatial distribution directly governs the efficiency and polarity of contact electrification. By leveraging this dynamic regulation, SL‐TENGs can serve as powerful probes for environmental information scavenging, enabling autonomous sensing of phase, temperature, and interfacial chemistry changes with high fidelity and without an external power supply.

### 4.2 Information Modulation Based on the Dynamic Regulation of the Entire EDL

Building upon the sensitive charge readout capabilities enabled by the dynamic regulation of the diffuse layer, the EDL itself, when viewed as a fully tunable interfacial system, offers a powerful platform not only for information scavenging but also for modulation. Beyond passive sensing, active manipulation of the EDL's structure and ionic composition enables complex behaviors such as signal gating, memory formation, and longer‐distance ionic communication. These capabilities bridge the gap between ionic sensing and adaptive response, laying the foundation for intelligent, energy‐autonomous systems. In this context, dynamic regulation of the entire EDL has emerged as a versatile mechanism for encoding, storing, and transmitting information at soft interfaces. By leveraging ion migration, surface charge redistribution, and interfacial polarization, recent studies have demonstrated the feasibility of constructing bioinspired neuromorphic circuits, emulating synaptic plasticity via ionic memory effects, and achieving underwater ionic signal transmission without external power supplies. These developments mark a shift from EDLs as passive sensing media to active information processing units, offering new possibilities for soft intelligence, bionic electronics, and self‐powered communication.

#### 4.2.1 Bioinspired Neuromorphic Circuit Control

The EDL serves not only as a fundamental structure for ionic‐electronic coupling but also as an active medium for mimicking neuromorphic information processing. Recent advances have demonstrated that by dynamically regulating the charge distribution across the EDL, particularly the diffuse and Stern layers, it is possible to construct bionic circuits that emulate neural signal propagation and coordination without relying on external power. These bioinspired neuromorphic circuits achieve lower‐power, direction‐sensitive control through triboiontronics strategies that manipulate charge type, polarity, and spatial configuration in response to mechanical or electrostatic stimuli.

**Figure 10:**
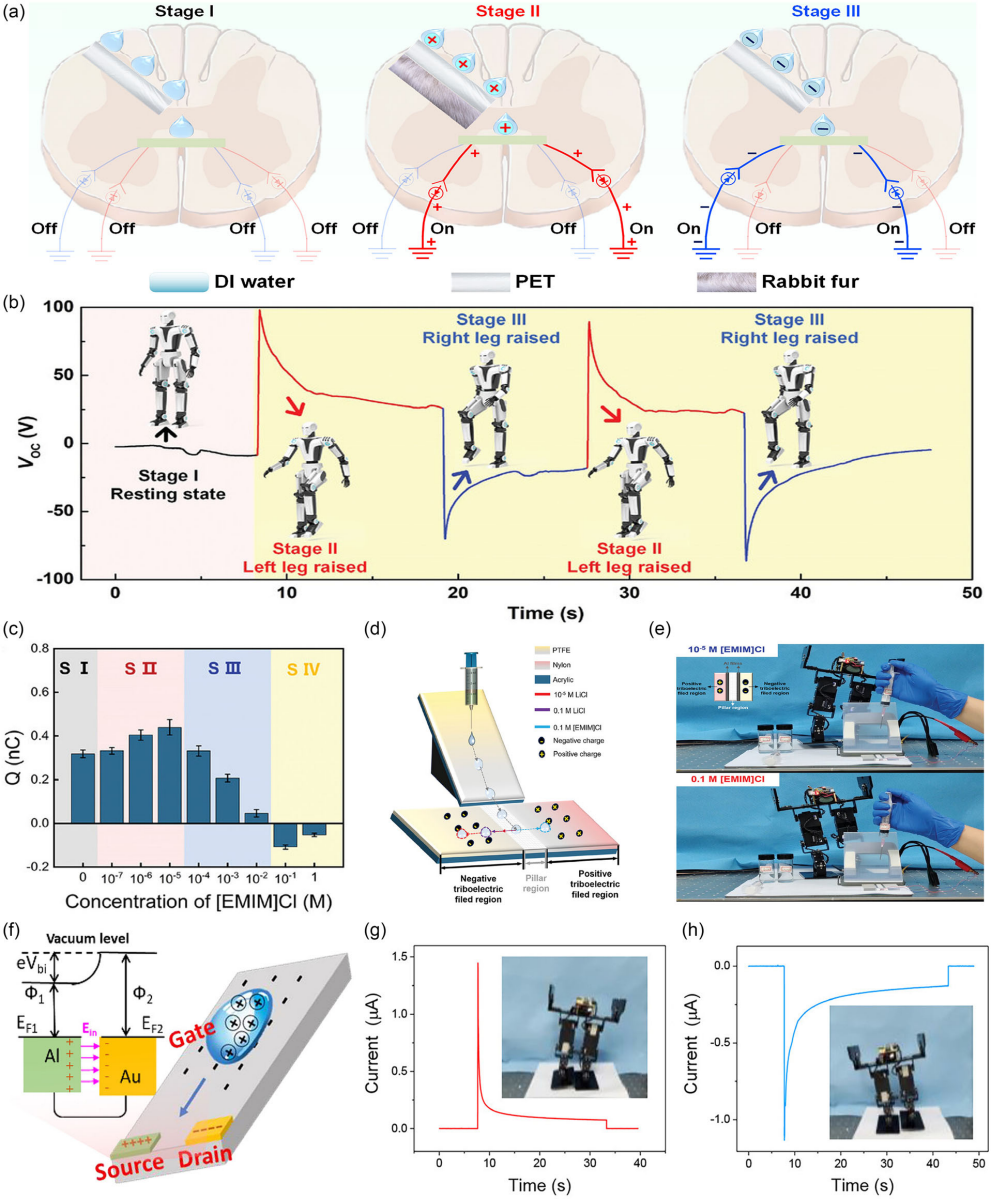
Bionic circuits were enabled by dynamic regulation of the EDL. a) Li et al. (2023) demonstrated that bidirectional triboelectric polarization could remotely modulate polarity switching in the diffuse layer and direction‐sensitive signal encoding. Reproduced with permission.^[^ ^80^ ^]^ Copyright 2023, Elsevier Inc. b) This mechanism mimics neuronal bidirectional control, enabling robotic actuation without external power. c,d) By tuning ion asymmetry and concentration in droplets, ionic logic, and voltage‐gated synapse‐like behaviors were realized by Li et al. in 2024.Reproduced with permission.^[^ ^102^ ^]^ Copyright 2024, Wiley‐VCH GmbH. e) These droplet‐electrode interactions emulated synaptic weight modulation and enable parallel, energy‐autonomous signal processing. f) Built‐in electric fields induced by metalwork function differences drove directional ion transport in water droplets, forming transistor‐like triboiontronics devices by Li et al. in 2024.Reproduced with permission.^[^ ^103^ ^]^ Copyright 2024, The Author(s). g,h) Such gated droplets mimic motor neuron activation, controlling rhythmic leg movement in soft robotics.

A representative work by Li et al. in 2023 explored how bidirectional triboelectric‐induced polarization, generated via solid‐solid contact electrification, could remotely regulate the charge distribution in the sub‐nanoconfined Stern layer, thereby switching the ionic polarity of the diffuse layer^[^ ^80^ ^]^ (**Figure** **10**a). This allowed the encoding of contact states into tunable ionic polarity and, consequently, into direction‐specific electronic outputs. The dynamically regulated EDL served as the core of a triboiontronics bioinspired neuromorphic circuit, in which water mist acted as a charge carrier. Depending on whether positively charged fur was applied or removed from a PET sprayer, the water droplets induced opposite‐polarity electronic currents. This polarity reversal directly mimicked the bidirectional neuronal control of limb movement, enabling LED switching and virtual robotic actuation without any external power (Figure 10b). Notably, the charge modulation process was non‐interfering and reversible, showcasing excellent control fidelity and robustness. This approach draws a compelling analogy with the synaptic function of biological neurons, where ion flow across membranes underpins signal directionality and excitation‐inhibition coordination. While not replicating biological action potentials in a strict sense, this triboelectric control of ionic polarization represents a bioinspired, self‐powered strategy for temporal signal encoding through ion gating. Compared to conventional neuromorphic systems, which require dedicated power and switching circuitry, our triboiontronic framework achieves low‐energy, self‐regulated signal modulation purely through physical contact, offering significant advantages for integration into soft robotics, neuroprosthetics, and biohybrid sensory systems. Furthermore, regarding the achievable temporal resolution of such programmable modulation, the upper limit is fundamentally constrained by ion migration dynamics. The onset of interfacial polarization is governed by electronic processes, specifically, electron cloud overlap and contact electrification, whose timescale is within 100 ps, as supported by ultrafast spectroscopy studies.^[^ ^110^, ^111^ ^]^ This ensures that the electric field driving ion transport can be established nearly instantaneously upon contact. However, the actual ionic response time is determined by the mobility and displacement of ions within the diffuse layer, which depends on both the ion species and the geometry of the interface. For example, assuming typical ion diffusion coefficients (10^−9^‐10^−11^ m^2^ s^−1^) and a migration distance of 10‐100 nm, the characteristic time for ion redistribution is estimated to be in the range of 1 μs to 1 ms. Therefore, while the electronic trigger for polarization switching is effectively instantaneous, the practical resolution for modulating ionic signals is typically in the sub‐millisecond to millisecond range. This is already compatible with many biological signaling processes, such as central pattern generators (CPGs), and is sufficiently fast for soft robotic actuation and bioelectronic interfaces.

Expanding beyond static regulation of surface charge, another study introduced a triboiontronics system where the ionic behavior of droplets was modulated through ion species and concentration. By controlling cation‐anion asymmetry in charged droplets, distinct ionic logic functions were realized by Li et al. in 2024^[^ ^102^ ^]^ (Figure 10c). For example, droplets containing different concentrations (10^−5^ or 10^−1^) of [EMIM]+ and Cl^−^ can differentially bridge electrode pairs, creating a voltage‐gated logic system similar to neuronal synapses (Figure 10d). Importantly, both the ionic identity and migration behavior modulated the transient state of the neuromorphic circuit. Through spatially patterned electrode arrays, these charged droplets could activate distinct logic branches, leading to a biomimetic neural gate capable of executing AND/OR logic depending on droplet composition and pathway. This dynamic droplet–electrode interaction closely resembles synaptic weight adjustment and enables parallel processing, offering an energy‐autonomous platform for in‐sensor computing and soft robotics (Figure 10e). Building upon this ionic behavior, a further enhancement was achieved by Li et al. in 2024,^[^ ^103^ ^]^ which leveraged the intrinsic work function difference between metal electrodes to establish a built‐in electric field. This field acted as a constant driving force for the directional migration of triboelectrically charged ions in water droplets, enabling transistor‐like triboiontronics devices (Figure 10f). The droplet served as a dynamic gate, and the work function mismatch between asymmetric electrodes (e.g., Al and Au) functioned analogously to a source‐drain junction. The synergistic effect between solid–liquid contact electrification and electrode‐induced electrochemical potential allowed for record‐high charge density (13.926 mC m^−2^) and stable DC outputs. More importantly, this configuration provided reliable threshold sensing with a high signal‐to‐noise ratio (SNR > 50 dB), enabling neuromorphic circuits that could distinguish movement direction, force magnitude, and spatial position. In a biomimetic demonstration, these gated droplets controlled the alternating motion of robotic legs, mimicking the rhythmic activation of motor neurons by excitatory and inhibitory signals (Figure 10g,h). The robust rectification and direction‐selective ionic control provided by the built‐in field offer an elegant analog to the potential gradient across neuronal membranes, crucial for spike generation and synaptic plasticity.

Collectively, these studies revealed a coherent strategy for constructing bioinspired neuromorphic circuits by harnessing the charge‐tunable features of EDLs. Whether through electrostatic field modulation, ionic species design, or electrode work function engineering, each approach exploited the EDL as a responsive, reconfigurable interface to bridge mechanical stimuli and electrical logic. By replacing traditional semiconductor‐based neuromorphic systems with fluidic, triboiontronics alternatives, these bioinspired architectures could not only achieve energy autonomy but also introduce new avenues for material intelligence, adaptive robotics, and human‐machine integration.

#### 4.2.2 Ionic Memory for Synaptic Emulation

The realization of bioinspired neuromorphic circuits based on EDL modulation demonstrates the feasibility of using ionic charge polarity and spatiotemporal electric field control to simulate real‐time neuronal signaling and decision‐making. However, beyond transient signal transmission, biological synapses also possess intrinsic memory characteristics, retaining stimulus history to shape subsequent responses. To emulate such synaptic plasticity, it is essential to transition from dynamic ionic regulation toward ionic memory behaviors, wherein the EDL not only mediates immediate charge transport but also encodes and preserves past input information. In this context, recent advances in polyelectrolyte‐confined fluidic systems offer a promising platform for constructing synapse‐like ionic devices capable of nonvolatile, history‐dependent response, bridging EDL‐based signal modulation with memory emulation in neuromorphic architectures.

**Figure 11:**
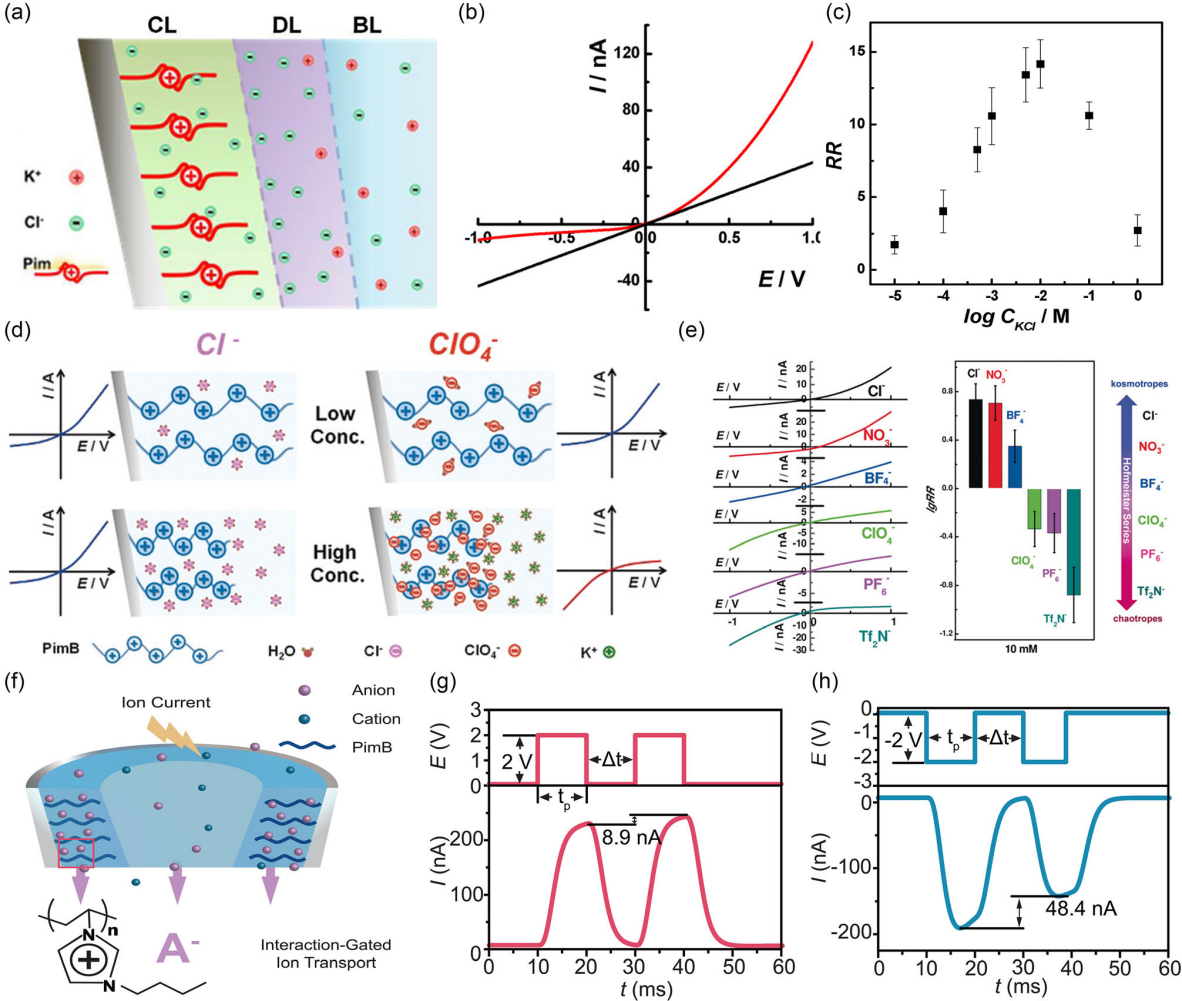
Ionic memory devices could be realized by dynamic regulation of the EDL. a,b) He et al. (2017) achieved ion current rectification in symmetric electrolytes using PimB‐modified micropipettes. Reproduced with permission.^[^ ^104^ ^]^ Copyright 2017, American Chemical Society. c) The rectification behavior was tunable via ion concentration. d,e) In 2018, rectification inversion was induced by chaotropic anions, revealing charge inversion in the interfacial EDL. Reproduced with permission.^[^ ^105^ ^]^ Copyright 2018, Wiley‐VCH Verlag GmbH & Co. KGaA, Weinheim. f) Xiong et al. developed a PFM in 2023. Reproduced with permission.^[^ ^106^ ^]^ Copyright 2023, The American Association for the Advancement of Science. g,h) The PFM exhibited pinched hysteresis and history‐dependent current, enabling short‐term plasticity and ionic‐electronic signal coupling.

To emulate the essential functions of biological synapses, especially the temporal plasticity and signal integration characteristics, ionic memory devices based on the dynamic regulation of EDLs have emerged as a compelling solution. These systems rely on the interplay between polyelectrolyte‐confined interfaces and ion‐selective transport behaviors, enabling the encoding and retention of electrical signals in a history‐dependent manner. In the foundational study of He et al. in 2017,^[^ ^104^ ^]^ a polyimidazolium brush (PimB) was grafted onto the inner walls of micropipettes to achieve micrometer‐scale ion current rectification in symmetric electrolytes (**Figure** **11**a,b). Despite the EDL thickness being much smaller than the channel radius, the asymmetric ion transport was induced by the space charge distribution within the PimB layer. This process was quantitatively interpreted via a three‐layer model comprising the charged layer, EDL, and bulk region, highlighting that sufficient interfacial charge density could enable rectification even beyond the nanometer regime. Importantly, the rectification behavior was tunable by varying polymer brush length, ion concentration (Figure 11c), and pipette geometry, providing a configurable platform for ionic logic operations with memory‐like behavior embedded in the charge redistribution process.

Building upon this framework, a subsequent study by He et al. in 2018 introduced chaotropic monovalent anion‐induced rectification inversion,^[^ ^105^ ^]^ further enriching the tunability of ionic behavior in the EDL on polyelectrolyte‐modified nanopipette surfaces (Figure 11d). The researchers discovered that certain anions (e.g., ClO_4_^−^) could over‐adsorb onto the PimB layer and reverse the direction of rectification, which is indicative of charge inversion in the EDL at the interface (Figure 11e). This concentration‐dependent phenomenon, modulated by both ion hydrophobicity (Hofmeister series) and brush hydrophobicity, effectively encodes chemical environment history into the interfacial ionic configuration. Such reversible and nonvolatile changes in rectification behavior formed the electrochemical basis for a memory function, akin to long‐term potentiation or depression in synapses. The culmination of these insights was embodied in the polyelectrolyte‐confined fluidic memristor (PFM) developed by Xiong et al. in 2023^[^ ^106^ ^]^ (Figure 11f). By integrating PimB‐confined conical fluidic channels with anion‐selective transport, the PFM device realized full memristive behavior in an aqueous environment. Its I–V response exhibited characteristic pinched hysteresis loops and history‐dependent current profiles, enabling not only short‐term plasticity but also chemical‐electric signal transduction (Figure 11g,h). For example, ClO_4_^−^ ions served as artificial neurotransmitters, modulating ion current spikes in analogy to synaptic transmission. Moreover, the PFM demonstrated latency and threshold behaviors under varying stimulus intensities, key elements of spike‐timing‐dependent plasticity. These findings validated that through careful engineering of polyelectrolyte‐ion interactions and spatial confinement, ionic memory can be reliably programmed and accessed, bridging the gap between chemical recognition and electrical signal output in neuromorphic systems.

Collectively, these studies formed a coherent progression: from establishing micrometer‐scale rectification via EDL asymmetry, to enabling ion‐specific and concentration‐dependent memory states, and finally to constructing biomimetic fluidic devices with genuine synaptic functionalities. This evolution could not only elucidate the fundamental roles of ion–surface interactions, steric confinement, and hydration energetics in shaping memory‐like behaviors, but also highlight the feasibility of encoding temporal information in purely ionic domains without requiring traditional electronic storage units. The dynamic modulation and retention of interfacial charge distributions in EDLs achieved through polyelectrolyte brushes, ion‐selective gating, and chaotropic ion effects laid the groundwork for constructing reconfigurable, multistate, and plastic ionic systems. Such fluidic synapses, with inherent signal‐dependent adaptability and biocompatibility, point toward a new generation of energy‐efficient, bio‐integrated neuromorphic computing platforms, where memory and computation are seamlessly embedded within the interface itself.

#### 4.2.3 Underwater Wireless Information Transmission

**Figure 12:**
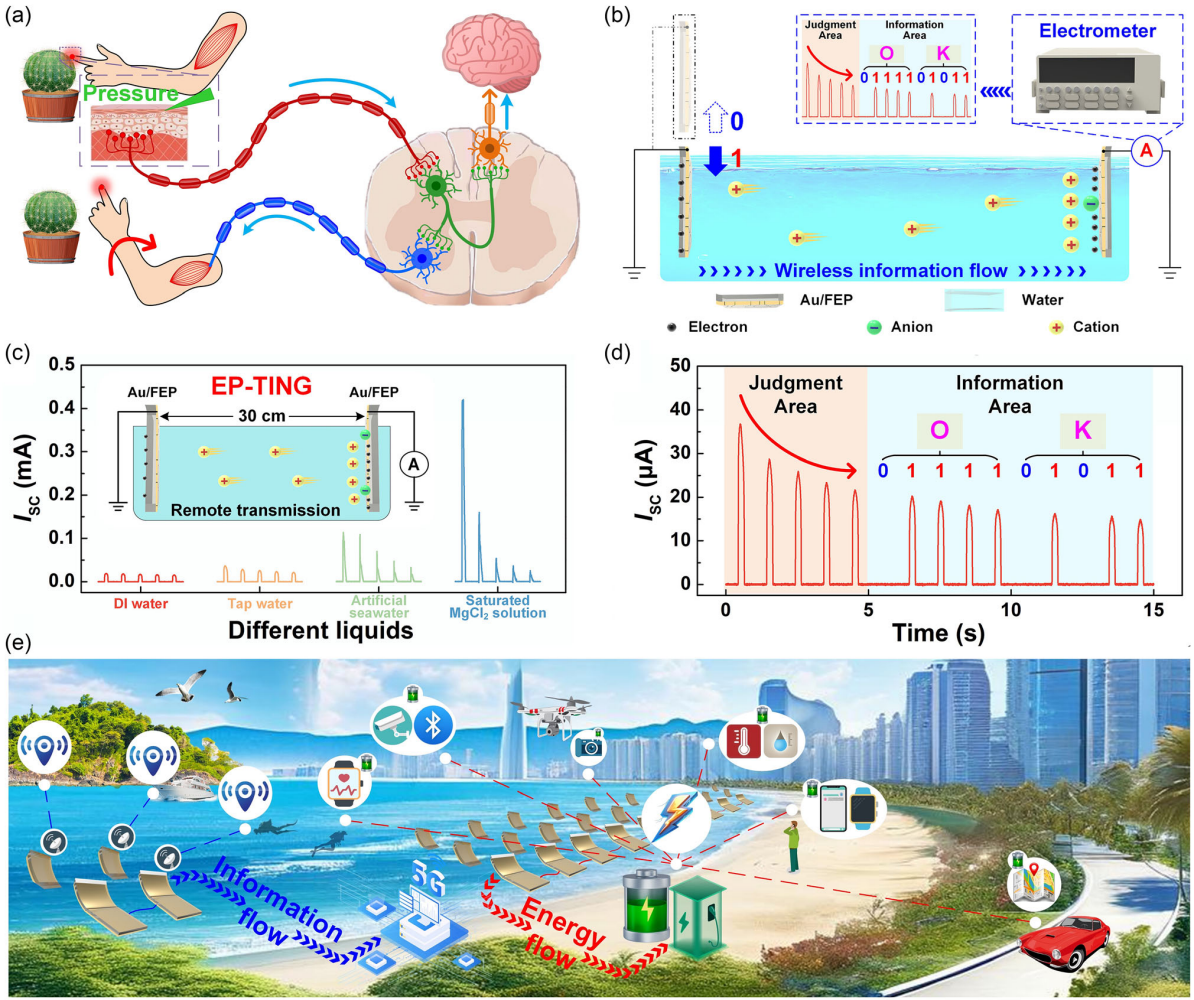
Underwater wireless information transmission could be realized by dynamic regulation of the EDL. a) In nervous systems, local ionic modulations underpin longer‐distance conduction of action potentials. Reproduced with permission.^[^ ^99^ ^]^ Copyright 2025, Elsevier Inc. b) In 2025, the EP‐TING developed by Li et al. could realize underwater wireless information transmission. c) It could achieve a wireless transmission distance of tens of centimeters. d) Information was encoded in the form of electrical pulses generated by mechanical actuation and decoded using the ASCII system. e) This paradigm could not only mirror biological neural communication but also advance the frontiers of in‐sensor computing and bioinspired IoT systems.

Building upon the ionic memory effects rooted in asymmetric EDL regulation, the potential of triboiontronic systems extends beyond static synaptic emulation toward dynamic signal propagation and spatially distributed communication, akin to biological neural networks. In nervous systems, memory and transmission are tightly coupled: local ionic modulations underpin not only synaptic plasticity but also the longer‐distance conduction of action potentials (**Figure** **12**a). Translating this principle, recent efforts have harnessed the same EDL‐based regulation strategies to construct bionic circuits capable of remotely relaying encoded ionic information across liquid media. By incorporating triboelectric‐induced polarization as a controllable driving force, these systems bridge the previously discrete domains of memory storage and information transfer, enabling wireless underwater communication that mimics the fidelity, directionality, and energy efficiency of biological signal transmission. This evolution marks a critical step from ionic memory toward fully integrated neuromorphic architectures with real‐time signal processing capabilities in aqueous environments.

A representative platform, the EP‐TING, based on asymmetric EDL enhanced by triboelectric‐induced polarization, was developed by Li et al. in 2025,^[^ ^99^ ^]^ integrating solid–solid contact electrification with ionic modulation at the solid–liquid interface (Figure 12b). The system comprised two Au/FEP hybrid films: One immersed in a liquid environment to maintain a stable EDL, and the other actuated to periodically engage in contact‐separation cycles. During operation, triboelectric‐induced polarization dynamically adjusted the surface potential of the dielectric substrate, effectively generating asymmetric EDLs across the two interfaces. This asymmetry established a concentration gradient that drives directed ion migration, extending the interaction distance well beyond the nanometer scale of a single EDL up to tens of centimeters in aqueous environments (Figure 12c). Analogous to signal propagation in biological nerve fibers, the triboiontronic Maxwell's demon, via the triboelectric‐induced polarization, enabled remote ionic transmission with higher fidelity and lower energy consumption. Information was encoded in the form of electrical pulses generated by mechanical actuation and decoded using the American Standard Code for Information Interchange (ASCII) system (Figure 12d). To minimize noise‐induced errors, a structured protocol defined the initial five pulses as a “judgment area” for source recognition, followed by an “information area” for content transmission. For instance, the character string “OK” was successfully transmitted and decoded via this process, demonstrating real‐time underwater signal transduction. To quantify performance, the system introduced a quality factor *Q* (A m^−1^), defined as the ratio of *I*_SC_ to the product of device area and transmission distance. The EP‐TING achieved a *Q* value of 12.0 A m^−1^, while its redox‐enhanced counterpart, the ES‐TING, reached 23.1 A m^−1^, surpassing conventional EDL‐based underwater transmitters, which were typically limited to *Q* values below 5 A m^−1^. These enhancements stemmed from the synergistic amplification of ion gradients by combining triboelectric‐induced polarization with electrochemical redox reactions. Therefore, addressing the fundamental limitations of traditional underwater communication methods, including electromagnetic attenuation, acoustic multipath interference, and optical absorption, triboiontronic systems based on asymmetric EDLs offered a compelling alternative.

In summary, underwater wireless information transmission via asymmetric EDLs presented a transformative approach to remote ionic signaling. Leveraging triboelectric‐induced polarization as triboiontronics Maxwell's demon, EP‐TINGs and ES‐TINGs achieved high signal‐to‐noise ratios, extended communication distances, and robust environmental adaptability within minimal device footprints. This paradigm could not only mirror biological neural communication but also pave the way toward integrated platforms that combine energy harvesting, signal transduction, and intelligent control, advancing the frontiers of in‐sensor computing and bioinspired Internet of Things (IoT) systems (Figure 12e).

### 4.3 The Neuromimetic Logic Gate Driven by Ionic‐Electronic Coupling Dynamics

Building upon the dynamic modulation of EDLs for both information sensing and encoding, the development of neuromimetic logic architectures represents a natural extension toward brain‐like computation. The above discussions have demonstrated how the temporal evolution of the diffuse layer enabled sensitive charge detection under dynamic interface conditions and how the entire EDL structure could be actively regulated for ionic polarity switching, ionic memory formation, and underwater wireless information transmission. These findings collectively suggest that beyond information flow, EDL‐driven systems are also capable of implementing logical operations, mimicking key features of neuronal computation such as signal integration, bidirectional modulation, and threshold‐controlled spike generation. In this context, it needs to explore how ionic‐electronic coupling dynamics, when strategically integrated with geometric and ionic asymmetry, can drive energy‐efficient, mechanoresponsive logic gates, paving the way for soft, self‐powered neuromorphic systems.

**Figure 13:**
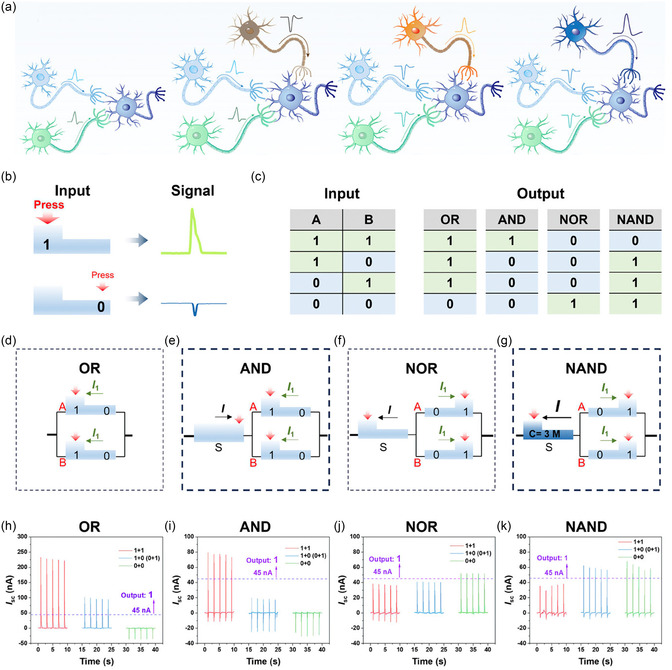
A mechanodriven hydrogel logic system was introduced by Ouyang et al. in 2025, which offered an innovative strategy to achieve logic‐level computation with minimal energy consumption. Reproduced with permission.^[^ ^109^ ^]^ Copyright 2025, Wiley‐VCH GmbH. a) The fundamental working principle of this system was inspired by axon‐mediated spike signal propagation in neurons. b) Input signals were assigned via pressure application on hydrogel regions, and logic outputs were determined by thresholding the resulting current amplitude. c) Truth table of four types of hydrogel logic gates. d–g) The structure of OR, AND, NOR, and NAND logic gates, respectively. h–k) *I*_SC_ of four hydrogel logic gates under different logic addition and subtraction.

Recent advances in neuromorphic engineering seek to emulate the lower‐energy, massively parallel computation capabilities of the human brain. Central to this paradigm is the implementation of ion‐based logic operations, wherein ionic charges, rather than electrons, carry, process, and store information. Ouyang et al. in 2025 introduced a mechano‐driven hydrogel logic system that mimics neuronal signal integration using piezoionic effects within geometrically asymmetric hydrogel iontronics, offering an innovative strategy to achieve logic‐level computation with minimal energy consumption.^[^ ^109^ ^]^ The fundamental working principle of this system was inspired by axon‐mediated spike signal propagation in neurons (**Figure** **13**a). When external pressure was applied to either the thick or thin side of a geometrically asymmetric PAAm hydrogel containing mobile ions, distinct ionic spike signals were generated. The direction and amplitude of the signal depended on both the applied pressure and the hydrogel's geometry. Thicker regions experienced higher localized stress, amplifying ion convection and generating higher amplitude positive signals, whereas thinner regions yielded weaker or even inverted (negative) signals. This polarity modulation enabled the encoding of binary states, with '1' and '0' represented by oppositely directed ionic spikes (Figure 13b,c).

The asymmetric geometry played a crucial role in enhancing force‐to‐electricity transduction. Finite element simulations confirmed that stress and strain are highly localized in the plateau region of the asymmetric hydrogel, leading to a higher Darcy flow velocity and improved ion mobility. More importantly, the selectivity of the 3D polymer network introduced differential mobility for anions and cations—a condition necessary for net ionic current generation. By varying the ion pair, not only the magnitude but also the direction of the ionic current could be modulated, effectively functioning as a polarity inverter for logic operations. Additionally, both higher salt concentration and lower actuation frequency were shown to enhance the amplitude of the ionic spike signal, mimicking synaptic plasticity and excitation/inhibition dynamics in biological systems. Harnessing these characteristics, the authors constructed four basic logic gates (OR, AND, NOR, NAND) using series‐parallel combinations of asymmetric hydrogels (Figure 13d‐g). Input signals were assigned via pressure application on hydrogel regions, and logic outputs were determined by thresholding the resulting current amplitude (Figure 13h‐k). For example, in the OR gate, either one or both hydrogels receiving input “1” generated a cumulative current exceeding the threshold, triggering an output “1”. Conversely, in the AND gate configuration, both hydrogels had to be activated simultaneously to overcome the inhibitory background signal and produce a logic‐high output. These logic gates were further applied in human–machine interaction tasks, such as controlling robotic arms, demonstrating real‐time response to tactile inputs. Notably, the spike signal generated by repeated actuation exhibited temporal decay and inversion, echoing the neurophysiological phenomena of synaptic depression and long‐term potentiation. This behavior could be harnessed for memory emulation, where the ionic signal evolution over time encodes a history of mechanical stimuli. In conclusion, the mechanodriven hydrogel logic gate system exemplified a novel neuromimetic computing paradigm that integrates tactile sensing, memory‐like behavior, and logic computation into a unified, self‐powered platform. The use of geometrically engineered hydrogels offered a scalable and biocompatible approach to artificial neural networks, while the piezoionic mechanism enabled dynamic modulation of ionic‐electronic coupling. This strategy laid the groundwork for next‐generation soft logic systems, including skin‐integrated processors, intelligent robotics, and wearable AI, where signal processing and actuation are seamlessly merged within an ionic medium.

Collectively, a unified strategy for solid–liquid interface information acquisition, modulation, and calculation could be achieved by systematically and dynamically regulating the EDL. Based on this, a progressive paradigm was illustrated: from scavenging latent interfacial information to modulating ion transport and encoding, and finally to executing logic operations in a biomimetic fashion. By leveraging the multiscale dynamics of EDL structures, these systems offer an energy‐autonomous, soft‐matter platform capable of sensing, computing, and communicating in environments where traditional electronics fail. This provides foundational insight for the development of next‐generation intelligent ionic‐electronic devices, especially in the emerging fields of neuromorphic engineering, flexible electronics, and bio‐integrated sensing networks.

## 5 Outlooks and Challenges

Building upon the diverse applications of dynamic regulation of EDLs in energy harvesting and information modulation, it becomes evident that the EDL serves as a unifying interfacial mechanism that bridges ionic‐electronic phenomena across both domains. Whether employed for amplifying energy conversion efficiency or enabling intelligent signal processing, the spatially and temporally tunable nature of EDLs offers a versatile platform for adaptive, responsive, and multifunctional devices. As research continues to evolve from proof‐of‐concept demonstrations toward practical implementation, it is essential to envision future directions that harness the full potential of EDL dynamics in real‐world scenarios. In this context, the following outlooks propose three key application frontiers for both energy devices^[^ ^150^, ^151^, ^152^, ^153^, ^154^, ^155^, ^156^, ^157^, ^158^, ^159^, ^160^, ^161^, ^162^, ^163^ ^]^ and information technologies,^[^ ^164^, ^165^, ^166^, ^167^, ^168^ ^]^ highlighting how EDL‐mediated interfacial control could reshape the design of next‐generation self‐powered and neuromimetic systems.

**Figure 14:**
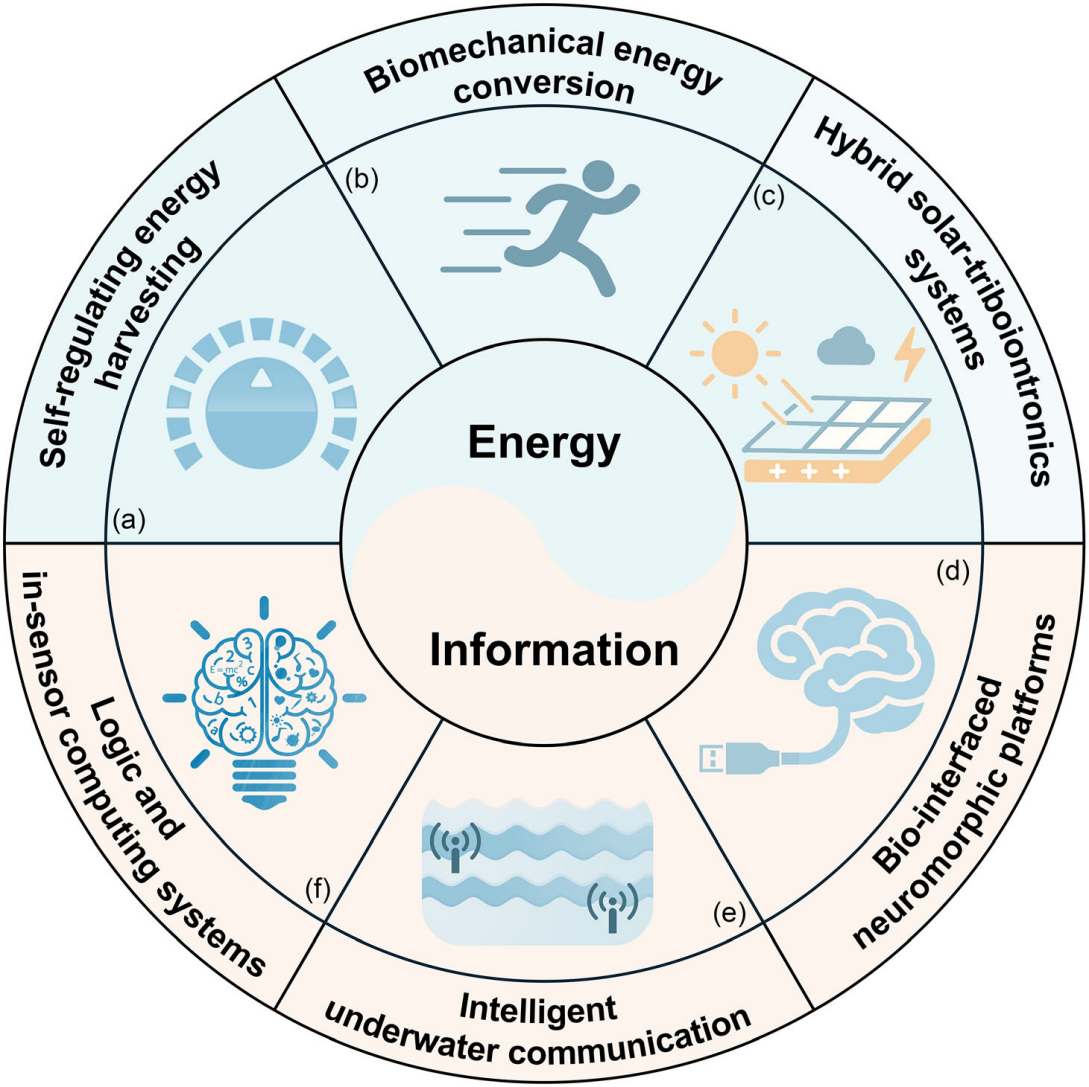
Prospects for the application of dynamic regulation of the EDL in the future energy and information fields. a) Self‐regulating energy harvesting. b) Biomechanical energy conversion. c) Hybrid solar‐triboiontronics systems. d) Bio‐interfaced neuromorphic platforms. e) Intelligent underwater communication. f) Logic and in‐sensor computing systems.

In the energy field, first, the dynamic regulation of the EDL offers a transformative strategy for constructing adaptive, multimodal energy interfaces (**Figure** **14**a). By coupling interfacial ion migration with external environmental cues, it is possible to realize self‐regulating energy harvesting systems that can dynamically adjust output based on local humidity, temperature, or chemical composition. Such systems, exemplified by LS‐TENGs and TINGs, hold strong potential for deployment in autonomous power platforms in harsh and variable environments, including environmental monitoring stations, ocean buoys, and field‐deployed sensor networks. Secondly, the inherent softness, lower‐frequency responsiveness, and liquid compatibility of EDL‐based systems make them highly attractive for biomechanical energy conversion applications (Figure 14b). In wearable or implantable bioelectronic systems, small‐scale physiological activities, such as sweating, fluid movement, or subtle body motions, can generate interfacial electric fields through contact electrification. These fields, when coupled with dynamically regulated EDLs, enable efficient ion–electron conversion, offering a viable strategy for physiological‐signal‐driven power supplies tailored to personalized medicine and continuous health monitoring. Thirdly, EDL dynamics also provide new opportunities for integrated hybrid energy systems, particularly those combining photovoltaic and triboelectric effects (Figure 14c). Through tribo‐induced polarization and work function modulation, interfacial energy level alignment and charge extraction in optoelectronic devices (e.g., perovskite solar cells) can be substantially enhanced. This approach enables the development of hybrid platforms capable of harvesting solar and mechanical energy simultaneously, offering a sustainable solution for energy‐autonomous electronics in smart buildings, portable devices, and multifunctional urban infrastructure.

In the information domain, firstly, the programmability and spatial specificity of EDLs open new frontiers for constructing neuromorphic biointerfaces capable of encoding, processing, and responding to complex ionic signals (Figure 14d). The ability to emulate synaptic plasticity, construct memristive neurons, and implement bionic logic operations through ionic‐electronic coupling underpins the design of hybrid systems for brain‐machine interfaces, soft robotics, and implantable neuroprosthetics. Such platforms are expected to support multichannel, bidirectional neural communication, enabling real‐time perception–processing–feedback integration in soft and dynamic environments. Second, the use of asymmetric EDLs with tunable ion gradients and polarization strength facilitates the development of intelligent underwater communication platforms (Figure 14e). By leveraging EDL dynamics to modulate signal polarity and transmission distance, such systems can overcome the inherent limitations of acoustic and optical underwater communication. This capability opens the door to constructing wearable, biomimetic communication networks for coordinated underwater operations, deep‐sea environmental monitoring, and interspecies biosignal detection, laying the groundwork for intelligent ocean networks. Third, in‐sensor computing architectures based on diffuse‐layer modulation promise to reshape the relationship between sensing and computation (Figure 14f). By enabling localized ion redistribution in response to environmental stimuli, sensors can directly perform logic operations and output decisions without relying on external processors. When integrated with flexible dielectrics, programmable liquid pathways, and bioinspired interfaces, these platforms will allow for real‐time, edge‐level intelligence, making them highly suitable for smart skins, IoT nodes, and distributed artificial intelligence systems.

Despite the transformative potential of dynamically regulated EDLs in enabling energy‐autonomous and information‐intelligent systems, several fundamental challenges remain to be addressed before practical, large‐scale deployment can be realized. First, the spatiotemporal precision of EDL regulation remains a fundamental bottleneck. Current triboiontronic architectures primarily rely on macroscale stimuli, such as global contact electrification, bulk ion concentration gradients, or mechanical contact, which lack the spatial selectivity and nanosecond‐level fast response capability required for high‐density, real‐time information encoding. Overcoming this limitation will likely require the development of localized and programmable actuation strategies. Emerging approaches such as optically triggered ion modulation, surface acoustic wave‐induced EDL tuning, or field‐effect control in confined nanochannels may offer promising routes toward rapid and spatially resolved EDL reconfiguration. Second, interface stability and signal reproducibility remain critical challenges under repeated mechanical cycling or ionic flux. The dynamic nature of the solid–liquid interface, particularly under continuous actuation or ionic accumulation, may lead to surface degradation, charge trapping, or hysteresis effects, ultimately compromising device reliability. To enhance operational longevity, future designs may benefit from incorporating self‐healing hydrogel supports, reversible ion‐binding sites, or stimuli‐responsive surface chemistries that preserve EDL fidelity under fluctuating environmental and mechanical loads. Third, the absence of integrated design frameworks for co‐optimizing energy harvesting and signal processing currently limits the system‐level performance of triboiontronic devices. For example, boosting interfacial polarization to maximize energy output can sometimes obscure subtle ionic variations essential for sensing or logic. Conversely, information‐centric tuning (e.g., selective ion gating) may reduce ionic mobility and degrade energy conversion efficiency. Addressing this trade‐off requires establishing a unified, multifunctional EDL design paradigm in which energy and information are synergistically encoded and transduced. Such frameworks could enable functionalities such as energy‐encoded signal transmission, feedback‐driven energy modulation, or computation‐coupled charge redistribution, essential features for future intelligent ionic‐electronic platforms. In summary, while significant advances have been made at the device level, overcoming the above challenges will be essential for realizing robust, adaptive, and scalable triboiontronic systems suited for real‐world applications.

## 6 Conclusion

The EDL at dielectric‐liquid interfaces has been established as a fundamental and versatile platform for coupling ionic and electronic processes, enabling both efficient energy harvesting and intelligent information modulation. Through a systematic revisit of its theoretical evolution, from the classical GCS model at conductor‐liquid interfaces to the passive two‐step EDL framework at nonconductor‐liquid interfaces, and ultimately to the actively reconfigurable triboiontronic paradigm tailored for nonconductive interfaces, its structural and functional progression has been elucidated, particularly under mechanical stimuli and in electronically insulating systems. In this review, the dynamic regulation of EDLs has been categorized based on targeted substructures, such as the diffuse layer or the entire EDL, and their translation into functional platforms has been demonstrated. In energy‐related applications, SL‐TENGs, hydrovoltaic nanogenerators, and different types of TINGs have been utilized to harness directional ion transport and interfacial polarization for mechanical or thermal energy conversion. In information‐related domains, dynamically modulated EDLs have been applied to interfacial charge probes, ionic memory formation, neuromorphic circuit control, and wireless communication, mimicking biological systems while operating under low‐power or energy‐autonomous conditions. Looking forward, EDL‐based iontronic systems are anticipated to underpin future intelligent technologies. However, several fundamental challenges remain to be addressed, necessitating interdisciplinary efforts in materials science, interfacial physics, and system‐level engineering. Collectively, this review highlights the transformative potential of actively regulated EDLs, not merely as electrochemical interfaces, but as foundational building blocks for multifunctional, adaptive platforms capable of seamless energy‐information coupling in the post‐Moore era.

## Conflict of Interest

The authors declare no conflict of interest.

## Author Contributions

**Xiang Li**: writing—original draft (lead) and writing—review and editing (lead). **Yu Wei**: writing—review and editing (lead). **Zhong Lin Wang**: conceptualization (lead). **Di Wei**: conceptualization (lead); writing—original draft (lead); and writing—review and editing (lead). **Xiang Li** and **Yu Wei** contributed equally to this work.
